# Antiviral Cyclopropane Acids from Deep-Sea-Derived Fungus *Aspergillus sydowii*

**DOI:** 10.3390/md20070410

**Published:** 2022-06-23

**Authors:** Siwen Niu, Shuhuan Huang, Bihong Hong, Qixi Huang, Xiupian Liu, Zongze Shao, Gaiyun Zhang

**Affiliations:** 1 Technology Innovation Center for Exploitation of Marine Biological Resources, Key Laboratory of Marine Genetic Resources, Third Institute of Oceanography, Ministry of Natural Resources, Xiamen 361005, China; niusiwen@tio.org.cn (S.N.); huangsh2268@163.com (S.H.); bhhong@tio.org.cn (B.H.); liuxiupian@tio.org.cn (X.L.); shaozongze@tio.org.cn (Z.S.); 2 Fangchenggang Center of Inspection and Testing, Fangchenggang 538000, China; fcgsjyjczx@163.com

**Keywords:** *Aspergillus sydowii*, deep-sea-derived fungus, cyclopropane, antiviral activities, H1N1

## Abstract

Four novel monocyclic cyclopropane acids, namely, sydocyclopropanes A–D (**1**–**4**), along with one known congener hamavellone B (**5**), were isolated from the *Aspergillus sydowii* MCCC 3A00324 fungus, which was isolated from the deep-sea sediment. The gross structures of novel compounds were established by detailed analyses of the spectroscopic data (HRESIMS and NMR spectra), and their absolute configurations were resolved on the basis of the quantum chemical calculations of ECD and NMR data, in association with DP4+ probability analyses. Sydocyclopropanes A–D, featuring the 1,1,2,3-tetrasubstituted cyclopropane nucleus with different lengthy alkyl side chains, were discovered in nature for the first time. All compounds exhibited antiviral activities against A/WSN/33 (H1N1), with IC_50_ values ranging from 26.7 to 77.2 μM, of which compound **1** exhibited a moderate inhibitory effect (IC_50_ = 26.7 μM).

## 1. Introduction

Cyclopropane is the smallest cycloalkane in chemistry. The strained cyclopropane subunits often occur in structurally complex natural products, especially in terpenoids, steroids, and alkaloids [1,2,3]. Many of them possess a wide range of biological activities, such as terpenoids euphorbactin and pre-schisanartanin with antiviral activities [4,5]; steroids cinanthrenol A, phrygiasterol, and klyflaccisteroid E with antitumor effects [6,7,8]; as well as alkaloids cottoquinazoline D and jawsamycin with antibacterial activities [9,10]. To date, thousands of natural products bearing cyclopropane moieties have been discovered in archaea, bacteria, fungi, and higher plants [2]. Most of them have polycyclic ring systems, while the monocyclic molecules are seldom found in nature. The (+)-*trans*-chrysanthemic acid was the first monocyclic cyclopropane isolated in 1920. Till now, a total of 15 metabolites bearing a monocyclic cyclopropane nucleus have been reported in nature, namely, hamavellones A and B [11], lysophosphatidylglycerol [12], methyl 9,10-methanohexadecanoate, methyl lactobacillate, methyl dihydrosterculate [13,14], plakoside A [15], 1-*O*-(*cis*-11′,12′-methyleneoctadecanoyl)-*sn*-glycero-3-phosphocholine [16], (2*S*,3*S*,4*R*)- and (2*S*,3*R*,4*S*)-2-(carboxycyclopropyl)glycine, (2*S*,3*S*,4*S*)-2-(carboxycyclopropyl)glycine [17], l-(2-acetoxyethyl)-2-hexylcyclopropane, l-(4-acetoxybutyl)-2-hexylcyclopropane, cascarillic acid, and 2-pentylcyclopropane carboxylic acid [18].

**Figure 1:**
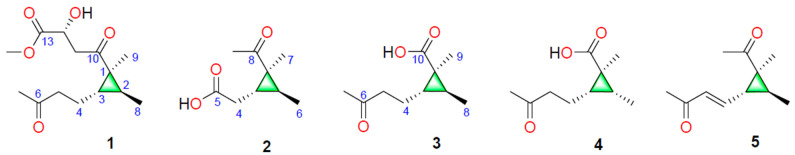
Chemical structures of the isolated metabolites **1**–**5**.

Our group mainly focused on finding new or bioactive metabolites from deep-sea-derived fungi [19,20,21,22,23]. Our previous chemical examination of the deep-sea sediment-derived fungus *Aspergillus sydowii* MCCC 3A00324 led to the isolation of undescribed sesquiterpenoids [24], monoterpenoids and polyketides [25], and acremolin alkaloids [26]. In our continuous efforts to find more new or active compounds, subsequent chromatography of the minor components of the fractions of the EtOAc extract of the fungus obtained four novel cyclopropane acids (sydocyclopropanes A–D, **1**–**4**) and one known congener hamavellone B (**5**) (Figure 1). Compounds **1**–**4** are the first representatives of single ring nucleus featuring 1,1,2,3-tetrasubstituted cyclopropane ring with different carbon side chains. All the isolated compounds were evaluated their antiviral effects against A/WSN/33 (H1N1), and **1**–**5** exhibited inhibitory effects with IC_50_ values ranging from 26.7 to 77.2 μM. Herein, the isolation, structural identification, and anti-H1N1 activities of **1**–**5** are presented.

## 2. Results and Discussion

**Figure 2:**
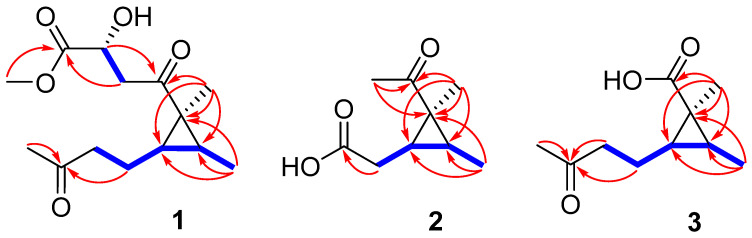
COSY (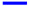) and key HMBC (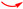) correlations of **1**–**3**.

Compound **1**, purified as a colorless oil, possessed the molecular formula C_14_H_22_O_5_, as deciphered on the basis of the HRESIMS spectrum at sodium adduct ion peak of *m*/*z* 293.1367 (calcd for C_14_H_22_O_5_Na, 293.1365), revealing four indices of hydrogen deficiency. The analysis of the ^1^H and HSQC spectra discovered three methyls (*δ*_H_ 0.98 (d, *J* = 5.8 Hz), 1.43 (s), and 2.16 (s)), one methoxyl (*δ*_H_ 3.73), three methylenes (*δ*_H_ 1.64 (m); 2.57 (t, *J* = 7.4 Hz); and 2.99 (dd, *J* = 17.8, 6.6 Hz), 3.14 (dd, *J* = 17.8, 4.3 Hz)), and three methines (4.51 (dd, *J* = 6.6, 4.3 Hz), 1.51 (q, *J* = 7.1 Hz), and 0.93 (m)) (Table 1). The ^13^C NMR spectrum exhibited 14 carbon resonance signals attributable to three carbonyl carbons (*δ*_C_ 175.9, 209.1, 211.5); one sp^3^ quaternary carbon (*δ*_C_ 36.9); three methines, including one oxygenated (*δ*_C_ 33.0, 34.9, 67.9); three methylenes (*δ*_C_ 24.1, 44.0, 46.2); three methyls (*δ*_C_ 12.3, 15.6, 30.0); and one methoxy group (*δ*_C_ 52.6) (Table 2). The aforementioned carbonyl functionalities accounted for 3 out of 4 degrees of unsaturation, revealing that **1** was featured a single ring framework. The COSY cross-peaks of H_2_-5 (*δ*_H_ 2.57)/H_2_-4 (*δ*_H_ 1.64)/H-3 (*δ*_H_ 1.51)/H-2 (*δ*_H_ 0.93)/H_3_-8 (*δ*_H_ 0.98), in association with the HMBC interactions from H_3_-8 to C-1 (*δ*_C_ 36.9), C-2 (*δ*_C_ 34.9), and C-3 (*δ*_C_ 33.0); from H_3_-9 (*δ*_H_ 1.43) to C-1/C-2/C-3/C-10 (*δ*_C_ 209.1); and from H_3_-7 (*δ*_H_ 2.16) to C-5 (*δ*_C_ 44.0) and C-6 (*δ*_C_ 211.5) deduced the presence of a cyclopropane ring, of which two methyl groups and a butan-2-one side chain were positioned at C-1, C-2, and C-3, respectively (Figure 2). Additionally, the COSY correlation between H_2_-11 (*δ*_H_ 2.99, 3.14) and H-12 (*δ*_H_ 4.51); together with the HMBC cross-peaks from H-12 to C-10, C-11 (*δ*_C_ 46.2), and C-13 (*δ*_C_ 175.9); as well as from OCH_3_ protons (*δ*_H_ 3.73) to C-13 determined the presence of a methyl 2-hydroxy-4-oxobutanoate side chain that connected to C-1. Thus, the planar structure of **1** was resolved as shown in Figure 1.

**Figure 3:**
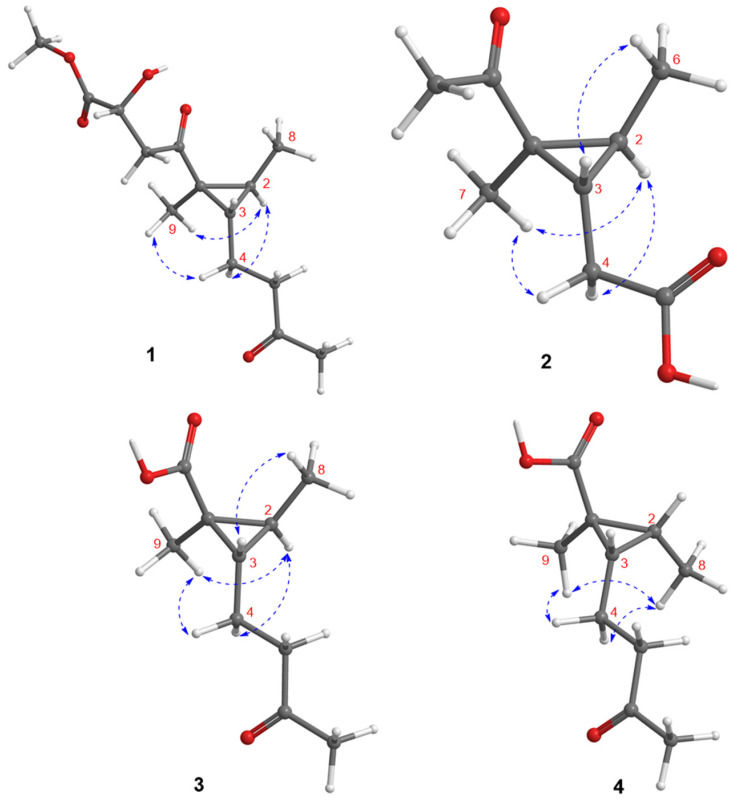
Key NOESY correlations of compounds **1**–**4**.

**Figure 4:**
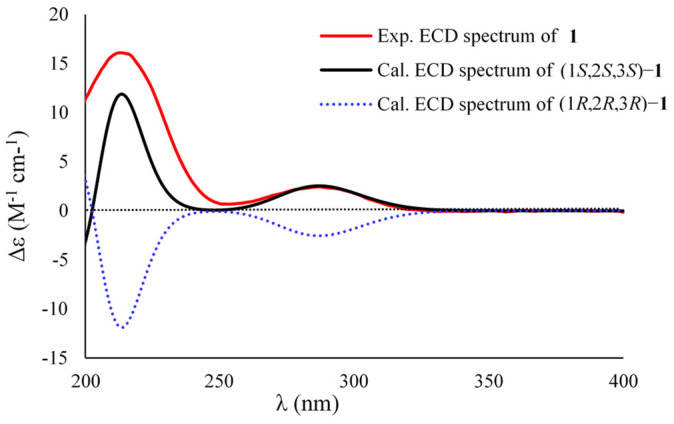
Experimental ECD spectrum of **1** in MeOH and the calculated ECD data of (1*S*,2*S*,3*S*)−**1** and (1*R*,2*R*,3*R*)−**1** at the B3LYP/6−311G(2d,p) level.

The relative configurations of C-1, C-2, and C-3 in the cyclopropane unit were assigned on the basis of the coupling constant and NOESY data. The coupling constant value of H-2 and H-3 (^3^*J*_H2,H-3_ = 7.1 Hz) indicated a *trans* relation of both protons. Moreover, the NOESY correlations from H_2_-4 to H-2 and H_3_-9 and from H_3_-9 to H-2 determined the 1S*, 2S*, and 3S* configurations (Figure 3). Noteworthily, the C-12 configuration was unresolved because it was resided at flexible side chain away from the cyclopropane moiety. In order to resolve the absolute configurations of the cyclopropane unit, the ECD calculated data of (1*S*,2*S*,3*S*)-**1** and its enantiomeric counterpart were obtained by the time-dependent density functional theory (TDDFT) method at the B3LYP/6-311G(2d,p)//B3LYP/6-311G(2d,p) level after a systematically conformational search with the OPLS3 force field [27]. Comparison of the calculated ECD data with that of the experimental one indicated the *S* configurations for C1, C-2, and C-3, respectively (Figure 4). Subsequently, the ^1^H and ^13^C NMR chemical shift calculations of the C-12 epimers (1*S*,2*S*,3*S*,12*R*)-**1** (**1a**) and (1*S*,2*S*,3*S*,12*S*)-**1** (**1b**) were carried out at the mPW1PW91/6-31+G(d,p) level in MeOH to relate the stereogenic relationships between C-12 and C-1/C-2/C-3. As a result, the calculated ^13^C NMR data of **1a** exhibited a better linear correlation coefficient (R^2^) value (R^2^ = 0.9984 for **1a** and 0.9969 for **1b**) and the lower root mean square error (RMSE) (RMSE = 1.88 for **1a** and 2.66 for **1b**), revealing the 12*R* configuration. The DP4+ probability analysis was also used to further establish the configuration, and **1a** showed the 100% probability to those of the experimental NMR data (Figure S29) [28], which further evidenced the above deduction. Therefore, **1** was determined as a novel molecule featuring a 1,1,2,3-tetrasubstituted cyclopropane nucleus, and given the name sydocyclopropane A.

**Figure 5:**
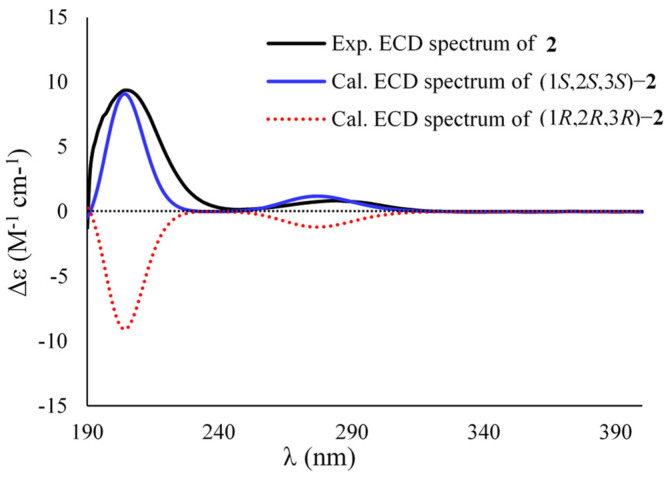
Experimental and calculated ECD spectra of **2**.

Sydocyclopropane B (**2**) has a molecular formula of C_9_H_14_O_3_ as established by the HRESIMS spectrum (*m*/*z* 193.0837, [M + Na]^+^) and ^13^C NMR data, indicating three degrees of unsaturation. The ^1^H NMR spectrum exhibited three methyls (*δ*_H_ 1.04, 1.43, and 2.27), one methylene (*δ*_H_ 2.35, 2.46), and two methines (*δ*_H_ 0.99 and 1.81), while the ^13^C NMR spectrum revealed nine carbon signals, including two carbonyl carbons (*δ*_C_ 176.6 and 211.2), one nonprotonated sp^3^ carbon (*δ*_C_ 36.5), two methines (*δ*_C_ 29.2 and 33.9), a methylene (*δ*_C_ 34.4), and three methyls (*δ*_C_ 12.0, 16.5, and 29.6). The above NMR data were similar to those of the co-existed hamavellone B (**5**), which was isolated from the soil fungus *Hamigera avellanea* BCC 17816 and then be totally synthesized to determine its absolute configuration [11,29], revealing a structurally related congener. The distinction was found that the side chain signals (two olefinic carbons, one ketone carbonyl, and a methyl) of **5** were replaced by one methylene (*δ*_H/C_ 2.35, 2.46/34.4) and a carbonyl carbon (*δ*_C_ 176.6) in **2**, indicating an acetic acid group located at C-3 (*δ*_C_ 29.2) in **2**. The assumption was evidenced by the COSY data from H-3 (*δ*_H_ 1.81) to H-2 (*δ*_H_ 0.99) and H_2_-4 (*δ*_H_ 2.35, 2.46) together with the HMBC correlations from H_2_-4 to C-1 (*δ*_C_ 36.5), C-2 (*δ*_C_ 33.9), C-3, and C-5 (*δ*_C_ 176.6) (Figure 2). The coupling constant ^3^*J*_H2,H-3_ (7.2 Hz) in association with the NOESY cross-peaks from H_2_-4 to H-2 and H_3_-7 (*δ*_H_ 1.43), from H_3_-7 to H-2, and from H_3_-6 (*δ*_H_ 1.04) to H-3 deduced the same relative configuration as that of **5** (Figure 3). The absolute configuration of **2** was determined to be the 1*S*, 2*S*, and 3*S* on the basis of comparison of their experimental and calculated ECD curves, as shown in Figure 5.

**Figure 6:**
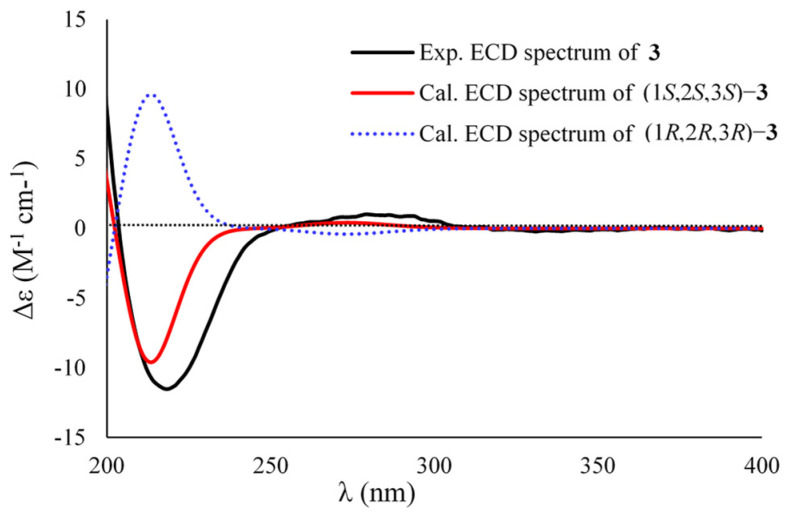
Experimental and calculated ECD data of **3**.

The molecular formula of sydocyclopropane C (**3**) was established to be C_9_H_14_O_3_ on the basis of the positive HRESIMS spectrum at *m*/*z* 207.0997 [M + Na]^+^ (calcd for C_10_H_16_O_3_Na, 207.0997), revealing three indices of hydrogen deficiency. The ^1^H and ^13^C NMR data of **3** resembled those of **1**, except that one ketone carbonyl, one methylene, an oxygenated methine, and methoxy signals in **1** disappeared in **3**. The HMBC interactions from H_3_-9 (*δ*_H_ 1.28) to C-1 (*δ*_C_ 29.2), C-2 (*δ*_C_ 31.5), C-3 (*δ*_C_ 32.8), and carbonyl carbon C-10 (*δ*_C_ 178.1) deduced a carboxylic acid group residing at C-1 in **3** instead of the methyl 2-hydroxy-4-oxobutanoate side chain of **1** (Figure 2). The relative configuration of **3** was uncovered to be the same as that of **1** on the basis of the coupling constants (^3^*J*_H2,H-3_ = 7.1 Hz) and NOESY cross-peaks from H-2 (*δ*_H_ 0.81) to H_2_-4 (*δ*_H_ 1.65) and H_3_-9, from H_3_-8 (*δ*_H_ 1.16) to H-3 (*δ*_H_ 1.36), and from H_2_-4 to H-2 (Figure 3). The similarly experimental ECD spectrum of **3** in methanol with the calculated ECD data of (1*S*,2*S*,3*S*)-**3** indicated the *S* configurations for C-1, C-2, and C-3, respectively (Figure 6).

**Figure 7:**
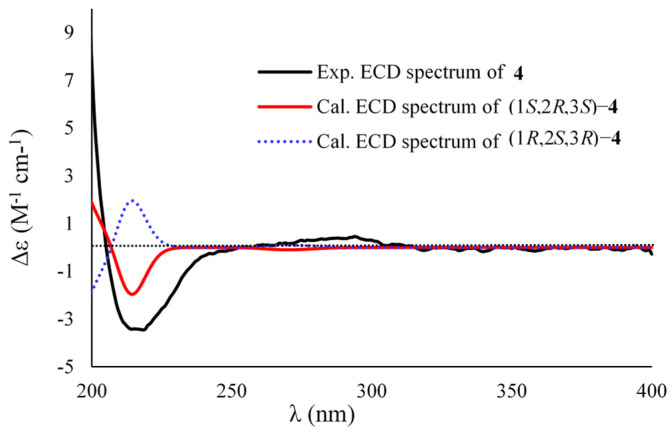
Experimental and calculated ECD spectra of **4**.

Compound **4** has the same molecular formula as that of **3**, as determined by the HRESIMS (*m*/*z* 207.1002, [M + Na]^+^) and ^13^C NMR spectra. The ^1^H and ^13^C NMR data were nearly identical to those of **3**, revealing a structurally similar analogue. The differences were attributed to the shielded chemical shifts of H-2 (Δ*δ*_H_ −0.72) and the deshielded H_3_-8 (Δ*δ*_H_ 0.13), C-1 (Δ*δ*_C_ 3.7), C-2 (Δ*δ*_C_ 7.2), C-8 (Δ*δ*_C_ 5.2), and C-9 (Δ*δ*_C_ 7.0) when compared with the corresponding NMR data of **3**. Analysis of the 2D NMR spectra of **4** established its gross structure to be identical to that of **3**. The large ^3^*J*_H2,H-3_ value (9.5 Hz) between H-2 and H-3, indicating the *cis* orientation of the vicinal protons, in association with the NOESY correlations from H_2_-4 (*δ*_H_ 1.59) to H_3_-8 (*δ*_H_ 1.03) and H_3_-9 (*δ*_H_ 1.15) and from H_3_-8 to H_3_-9 deduced its relative configurations to be 1S*, 2R*, and 3S* (Figure 3). In addition, the experimental ECD data of **4** matched well with the calculated ECD data of (1*S*,2*R*,3*S*)-**4**, indicating the *S* configurations for C-1 and C-3, and *R* for C-2 (Figure 7). Therefore, the structure of **4** was assigned as a C-2 epimer of **3** and named sydocyclopropane D.

Apart from compounds **1**–**4**, one known metabolite was obtained and established to be hamavellone B (**5**) on the basis of the comparison of the NMR data with those reported in the literature [11,29].

All the isolated compounds were evaluated the anti-influenza virus A/WSN/33 (H1N1) activities using the cytopathic effect (CPE) reduction assay [30], and oseltamivir (OSV) was used as the positive control. Cytotoxic evaluation was carried out via CellTiter-Glo assay to determine whether the antivirus effects was due to the toxicity of the tested compounds against MDCK cells. As a result, all the isolated compounds exhibited no cytotoxic activities at the concentration of 100 μM, and compounds **1**–**5** showed inhibitory H1N1 effects with IC_50_ values ranging from 26.7 to 77.2 μM (Table 3). Preliminary analysis of the structure–activity relationships found that the methyl 2-hydroxy-4-oxobutanoate side chain residing at C-1 in **1** significantly enhanced the antiviral activity, as evidenced by **1** exhibiting an IC_50_ value of 26.7 μM, while **3** showed 77.2 μM. Additionally, the C-3 chiral center had little effect on anti-H1N1 activities as exemplified by the C-3 epimers of **3** (IC_50_ = 77.2 μM) and **4** (IC_50_ = 66.4 μM).

## 3. Materials and Methods

### 3.1. General Experimental Procedures

Optical rotation data were recorded on the basis of the Anton Paar MCP 500 automatic polarimeter. The UV and ECD spectra were measured by the Shimadzu UV-1800 spectrophotometer and chirascan CD spectrometer, respectively. The Bruker Avance-400 FT NMR spectrometer was used to measure the NMR data. Chemical shifts (*δ*) were referenced to the CD_3_OD at 3.31 and 49.00 ppm for proton and carbon, respectively. The Xevo G2 Q-TOF mass spectrometer was used to record the HRESIMS spectra. Silica gel, sephadex LH-20, and ODS-A were used for column chromatography (CC). All solvents used for CC were analytical grade. Precoated silica gel plates were used for the thin-layer chromatography (TLC) analysis, and the TLC spots were visualized by heating the plates when sprayed with vanillin sulfuric acid chromogenic reagent. Semipreparative HPLC was performed on a Alltech LS class pump equipped with UV/Vis detector using YMC packed ODS-A (250 × 10 mm, 5 μm) column for the purification.

### 3.2. Fungal Strain, Identification, and Fermentation

The fungal strain was isolated from the deep-sea sediment (−2246 m) sampled from the South Atlantic Ocean (13.6639° W, 14.2592° S) in April 2011, and was identified as *Aspergillus sydowii* on the basis of the amplified internal transcribed spacer (ITS) gene sequence analysis (GenBank accession no. MN918102). The fungus was deposited at the Marine Culture Collection of China, Third Institute of Oceanography, Ministry of Natural Resources, Xiamen, China, and assigned the accession no. MCCC 3A00324. For chemical investigations, fresh mycelia and spores were cultured on PDA medium under 25 °C for 4 days, and then were inoculated into 2 × 500 mL Erlenmeyer flasks with PDB medium under rotary culture 4 days (200 rpm, 25 °C) to obtain seed cultures. Scale-up fermentation was performed on rice solid medium in 30 Erlenmeyer flasks, each containing rice (80 g) and sea water (120 mL). After autoclaving, each flask was inoculated into 3 mL seed cultures and cultured in static conditions for 26 days at 25 °C.

### 3.3. Extraction, Isolation, and Purification

The fermented substrate was extracted three times using ethyl acetate (EtOAc). The combined EtOAc was concentrated under reduced pressure to yield 16.2 g extract. The EtOAc extract was subjected to CC on silica gel vacuum liquid chromatography (VLC), eluting with increasing polarity from CH_2_Cl_2_ to MeOH (1:0~0:1) to yield two fractions (A and B). Fraction B (8.5 g) was chromatographed via ODS CC with MeOH/H_2_O gradient elution (30%~100%) to obtain fourteen subfractions (SF1–SF14). Subfraction SF3 (147 mg) was fractionated on the basis of the CC over silica gel, using petroleum ether (PE) and EtOAc isocratic elution (10:1), and then further purified by semipreparative HPLC eluted with 30% MeOH in H_2_O to obtain **2** (8.1 mg). Compound **1** (2.5 mg) was obtained from subfraction SF4 (151.9 mg) by CC over Sephadex LH-20 (CH_2_Cl_2_/MeOH, 1:1) and semipreparative HPLC (MeCN/H_2_O, 23:67). Subfraction SF5 (333.4 mg) was subjected to silica gel CC eluting with CH_2_Cl_2_/MeOH (30:1) to yield five fractions (SF5-1~SF5-5). Subfraction SF5-1 was purified by semipreparative HPLC with a mobile phase of 19% MeCN in H_2_O to obtain **2** (1.2 mg), while subfraction SF5-5 was subjected to semipreparative HPLC (MeCN/H_2_O, 7:18) to furnish compounds **3** (6.2 mg) and **4** (1.8 mg).

Sydocyclopropane A (**1**): colorless oil; ${\left[\alpha \right]}_{D}^{25}$ +46 (*c* 0.1, MeOH); UV (MeOH) *λ*_max_ (log *ε*) 207 (0.34) nm; ECD (MeOH) *λ*_max_ (Δ*ε*) 214 (+16.09), 289 (+2.37) nm; ^1^H and ^13^C NMR data, Table 1 and Table 2; HRESIMS *m*/*z* 293.1367 [M + Na]^+^ (calcd for C_14_H_22_O_5_Na, 293.1365).

Sydocyclopropane B (**2**): colorless oil; ${\left[\alpha \right]}_{D}^{25}$ +64 (*c* 0.32, MeOH); UV (MeOH) *λ*_max_ (log *ε*) 206 (0.49) nm; ECD (MeOH) *λ*_max_ (Δ*ε*) 206 (+9.34), 285 (+0.83) nm; ^1^H and ^13^C NMR data, Table 1 and Table 2; HRESIMS *m*/*z* 193.0837 [M + Na]^+^ (calcd for C_9_H_14_O_3_Na, 193.0841).

Sydocyclopropane C (**3**): colorless oil; ${\left[\alpha \right]}_{D}^{25}$ +13 (*c* 0.43, MeOH); ECD (MeOH) *λ*_max_ (Δ*ε*) 219 (−11.51) nm; ^1^H and ^13^C NMR data, Table 1 and Table 2; HRESIMS *m*/*z* 207.0997 [M + Na]^+^ (calcd for C_10_H_16_O_3_Na, 207.0997).

Sydocyclopropane D (**4**): colorless oil; ${\left[\alpha \right]}_{D}^{25}$ +10 (*c* 0.16, MeOH); ECD (MeOH) *λ*_max_ (Δ*ε*) 217 (−3.43) nm; ^1^H and ^13^C NMR data, Table 1 and Table 2; HRESIMS *m*/*z* 207.1002 [M + Na]^+^ (calcd for C_10_H_16_O_3_Na, 207.0997).

### 3.4. Anti-Influenza Virus H1N1 Assay

The anti-influenza virus test was carried out as previously reported [30], and influenza A/WSN/33 (H1N1) was used in the study. In brief, Madin–Darby canine kidney (MDCK) cells were cultured in Dulbecco’s modified Eagle medium (DMEM) (Gibco BRL, Inc., Gaithersburg, MD, USA) and supplemented with 1% fetal bovine serum (FBS) (PAA Laboratories, Linz, Austria) at 37 °C in 5% CO_2_. The MDCK cells were seeded into 96-well plates (1 × 10^5^ cells per well) and incubated for 24 h. Then, the cells were infected with influenza virus A/WSN/33 (multiplicity of infection, MOI = 0.1) and suspended in DMEM supplemented with 1% FBS, the test compounds, and 2 mg/mL TPCK-treated trypsin, with a final DMSO concentration of 1% in each well. After 40 h incubation, CellTiter-Glo reagent (Promega Corp., Madison, WI, USA) was added and the plates were then read using a plate reader (Tecan Infinite M2000 PRO™; Tecan Group Ltd., Mannedorf, Switzerland). Positive control was chosen for oseltamivir (OSV). The IC_50_ values were obtained using the Sigma Plot Statistical Analysis software as the test compound concentration required inhibiting cytopathic production after post-infection by 50%.

### 3.5. Cytotoxicity Test

The MDCK cells were grown in DMEM, supplemented with 1% FBS under a humidified atmosphere (5% CO_2_) at 37 °C. Cell suspension was placed into 96 well microtiter plates and incubated for 24 h. Then, the increasing amounts of test compounds were added to each well and further incubated for 40 h. Cytotoxicity was assessed with the CellTiter-Glo assay, as described above.

### 3.6. Computational Method

#### 3.6.1. NMR Calculation of Compound **1**

Conformational searches of (1*S*,2*S*,3*S*,12*R*)-**1** and (1*S*,2*S*,3*S*,12*S*)-**1** were undertaken by the Maestro 10.2 program with the OPLS3 molecular mechanics force within an energy window of 5.0 kcal/mol. The searches discovered 105 conformers for (1*S*,2*S*,3*S*,12*R*)-**1** and 133 conformers for (1*S*,2*S*,3*S*,12*S*)-**1**, which were optimized using density functional theory (DFT) at the B3LYP/6-31G(d) level in gas phase using the Gaussian 09 program. The optimized conformers, whose Boltzmann distributions of Gibbs free energies were more than 2%, were selected for the ^1^H and ^13^C NMR calculations using the GIAO method at the mPW1PW91/6-31G +(d,p) level in methanol with the PCM model. The DP4+ probabilities of (1*S*,2*S*,3*S*,12*R*)-**1** and (1*S*,2*S*,3*S*,12*S*)-**1** were analyzed by using the Excel spreadsheet, which was provided by Ariel M. Sarotti et al., to determine the C-12 configuration of **1**.

#### 3.6.2. ECD Calculations of Compounds **1**–**4**

The conformational searches of model structures **1**–**4** were performed according to the above NMR calculation description. Searched conformers were further optimized at the B3LYP/6-311G(2d,p) level in the gas phase by Gaussian 09 program. The optimized conformers with a Boltzmann population over 1% were chosen for the ECD calculations. The energies, oscillator strengths, and rotational strengths of the first 60 electronic excitations were calculated at the B3LYP/6-311G(2d,p) level in methanol with the conductor-like polarizable continuum model (CPCM). The calculated ECD data of the conformers were combined on the basis of their weighing the Boltzmann distribution rate using SpecDis 1.71 software [31], and Gaussian function band shape sigma was set as 0.3 eV.

## 4. Conclusions

In conclusion, chemical examination of the fermented cultures of the deep-sea sediment-derived *Aspergillus sydowii* MCCC 3A00324 fungus resulted in the isolation of four novel cyclopropane acids, named sydocyclopropanes A–D (**1**–**4**), together with one known congener hamavellone B (**5**). The novel structures were resolved on the basis of spectroscopic analysis (NMR and HRESIMS data) and ECD and NMR calculations, in association with DP4+ probability analyses. Compounds **1**–**4** are the first representatives of monocyclic ring systems featuring a 1,1,2,3-tetrasubstituted cyclopropane ring bearing different alkyl side chains, revealing deep-sea-derived fungi are new source of structurally novel compounds. All isolated metabolites were evaluated the anti-H1N1 effects by the CPE reduction assay, and **1–5** exhibited inhibitory activities with IC_50_ values ranging from 26.7 to 77.2 μM. Among them, compound **1** showed moderately antiviral activity (IC_50_ = 26.7 μM). This study is the first to discover single ring cyclopropane acids in marine-derived fungi, and more importantly, sydocyclopropanes exhibiting anti-H1N1 effects are reported here for the first time.

## Figures and Tables

**Table 1:** ^1^H NMR spectroscopic data of **1**–**4** recorded at 400 MHz in CD_3_OD (*δ* in ppm, *J* in Hz).

No.	1	2	3	4
2	0.93, m	0.99, m	0.81, m	1.53, m
3	1.51, q (7.1)	1.81, q (7.2)	1.36, q (7.1)	1.43, dt (9.5, 7.3)
4	1.64, m	2.46, dd (16.7, 7.3); 2.35, dd (16.7, 7.7)	1.65, m	1.59, m
5	2.57, t (7.4)		2.60, t (7.3)	2.58, t (7.5)
6		1.04, d (5.6)		
7	2.16, s	1.43, s	2.17, s	2.17, s
8	0.98, d (5.8)		1.16, d (6.2)	1.03, d (6.5)
9	1.43, s	2.27, s	1.28, s	1.15, s
11	3.14, dd (17.8, 4.3); 2.99, dd (17.8, 6.6)			
12	4.51, dd (6.6, 4.3)			
OCH_3_	3.73, s			

**Table 2:** ^13^C NMR spectroscopic data of **1**–**4** in CD_3_OD (100 MHz).

No.	1	2	3	4
1	36.9, C	36.5, C	29.2, C	25.5, C
2	34.9, CH	33.9, CH	31.5, CH	24.3, CH
3	33.0, CH	29.2, CH	32.8, CH	29.7, CH
4	24.1, CH_2_	34.4, CH_2_	24.1, CH_2_	18.7, CH_2_
5	44.0, CH_2_	176.6, C	44.0, CH_2_	43.7, CH_2_
6	211.5, C	12.0, CH_3_	211.5, C	211.3, C
7	30.0, CH_3_	16.5, CH_3_	29.9, CH_3_	29.9, CH_3_
8	12.3, CH_3_	211.2, C	13.0, CH_3_	7.8, CH_3_
9	15.6, CH_3_	29.6, CH_3_	15.7, CH_3_	8.7, CH_3_
10	209.1, C		178.1, C	180.7, C
11	46.2, CH_2_			
12	67.9, CH			
13	175.9, C			
OCH_3_	52.6, CH_3_			

**Table 3:** Inhibitory activities of **1**–**5** against influenza virus A/WSN/33 (H1N1).

Compounds	IC_50_ (μM)	CC_50_ (μM)
**1**	26.7 ± 0.9	>100
**2**	29.5 ± 1.4	>100
**3**	77.2 ± 0.5	>100
**4**	66.4 ± 1.7	>100
**5**	35.8 ± 3.2	>100
OSV	18.1 ± 1.2	>100

## Data Availability

Not applicable.

## Supplementary Materials

The following supporting information can be downloaded at: https://www.mdpi.com/article/10.3390/md20070410/s1, Figures S1–S28: HRESIMS, ^1^H, ^13^C, HSQC, COSY, HMBC, and NOESY spectra of novel structures **1**–**4**; Figure S29: DP4^+^ probability analyses of (1*S*,2*S*,3*S*,12*R*)-**1** and (1*S*,2*S*,3*S*,12*S*)-**1** at the mPW1PW91/6-31+G(d,p) level.
