# Supplementary material for: Antiviral Cyclopropane Acids from Deep-Sea-Derived Fungus Aspergillus sydowii
Source: Mar Drugs. 2022 Jun 23;20(7):410. doi: 10.3390/md20070410 (PMC9321810; doi:10.3390/md20070410)
Supplement: Supplementary file 1 [file marinedrugs-20-00410-s001.zip › marinedrugs-1773871-supplementary.pdf]

Supporting Information

Antiviral Cyclopropane Acids from Deep-Sea-Derived Fungus *Aspergillus sydowii*

Siwen Niu [1], Shuhuan Huang [1], Bihong Hong [1], Qixi Huang [2], Xiupian Liu [1], Zongze Shao [1] and Gaiyun Zhang [1,*]

[1] Technology Innovation Center for Exploitation of Marine Biological Resources, Key Laboratory of Marine Genetic Resources, Third Institute of Oceanography, Ministry of Natural Resources, Xiamen 361005, People's Republic of China; niusiwen@tio.org.cn (S.N.); huangsh2268@163.com (S.H.); bhhong@tio.org.cn (B.H.); liuxiupian@tio.org.cn (X.L.); shaozongze@tio.org.cn (Z.S.)

[2] Fangchenggang Center of Inspection and Testing, Fangchenggang 538000, People's Republic of China; fcgsjyjczx@163.com (Q.H.)

* Correspondence: zhgyun@tio.org.cn (G.Z.)

Contents　　Pages

Figure S1. HRESIMS spectrum of **1**. S3
Figure S2. ^{1}H NMR spectrum of **1** in CD_3OD (400 MHz). S4
Figure S3. ^{13}C NMR spectrum of **1** in CD_3OD (100 MHz). S5
Figure S4. HSQC spectrum of **1** in CD_3OD. S6
Figure S5. COSY spectrum of **1** in CD_3OD. S7
Figure S6. HMBC spectrum of **1** in CD_3OD. S8
Figure S7. NOESY spectrum of **1** in CD_3OD. S9
Figure S8. HRESIMS spectrum of **2**. S10
Figure S9. ^{1}H NMR spectrum of **2** in CD_3OD (400 MHz). S11
Figure S10. ^{13}C NMR spectrum of **2** in CD_3OD (100 MHz). S12
Figure S11. HSQC spectrum of **2** in CD_3OD. S13
Figure S12. COSY spectrum of **2** in CD_3OD. S14
Figure S13. HMBC spectrum of **2** in CD_3OD. S15
Figure S14. NOESY spectrum of **2** in CD_3OD. S16
Figure S15. HRESIMS spectrum of **3**. S17
Figure S16. ^{1}H NMR spectrum of **3** in CD_3OD (400 MHz). S18
Figure S17. ^{13}C NMR spectrum of **3** in CD_3OD (100 MHz). S19
Figure S18. HSQC spectrum of **3** in CD_3OD. S20
Figure S19. COSY spectrum of **3** in CD_3OD. S21
Figure S20. HMBC spectrum of **3** in CD_3OD. S22
Figure S21. NOESY spectrum of **3** in CD_3OD. S23
Figure S22. HRESIMS spectrum of **4**. S24
Figure S23. ^{1}H NMR spectrum of **4** in CD_3OD (400 MHz). S25
Figure S24. ^{13}C NMR spectrum of **4** in CD_3OD (100 MHz). S26
Figure S25. HSQC spectrum of **4** in CD_3OD. S27
Figure S26. COSY spectrum of **4** in CD_3OD. S28
Figure S27. HMBC spectrum of **4** in CD_3OD. S29
Figure S28. NOESY spectrum of **4** in CD_3OD. S30
Figure S29. DP4+ probability analyses of (1*S*,2*S*,3*S*,12*R*)-**1** and (1*S*,2*S*,3*S*,12*S*)-**1** at the mPW1PW91/6-31+G(d,p) level. S31

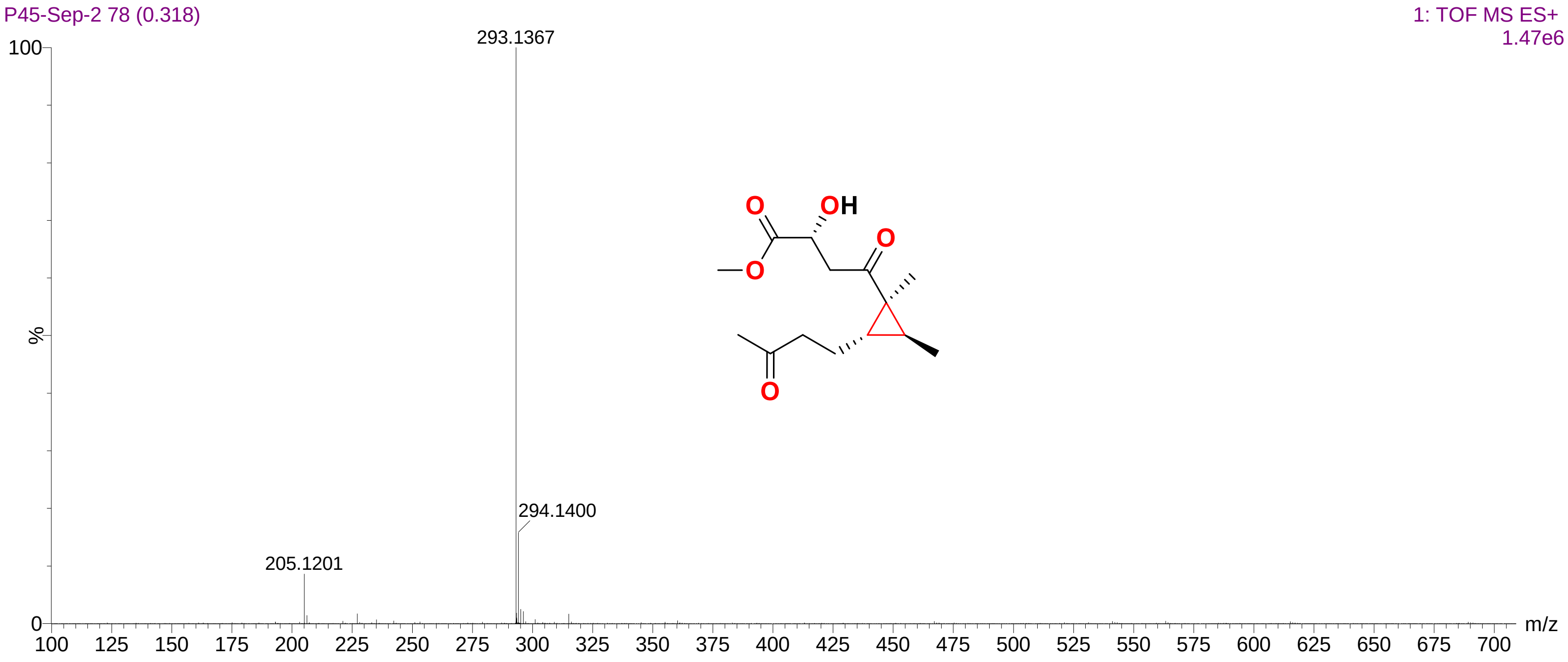

Figure S1. HRESIMS spectrum of **1**.

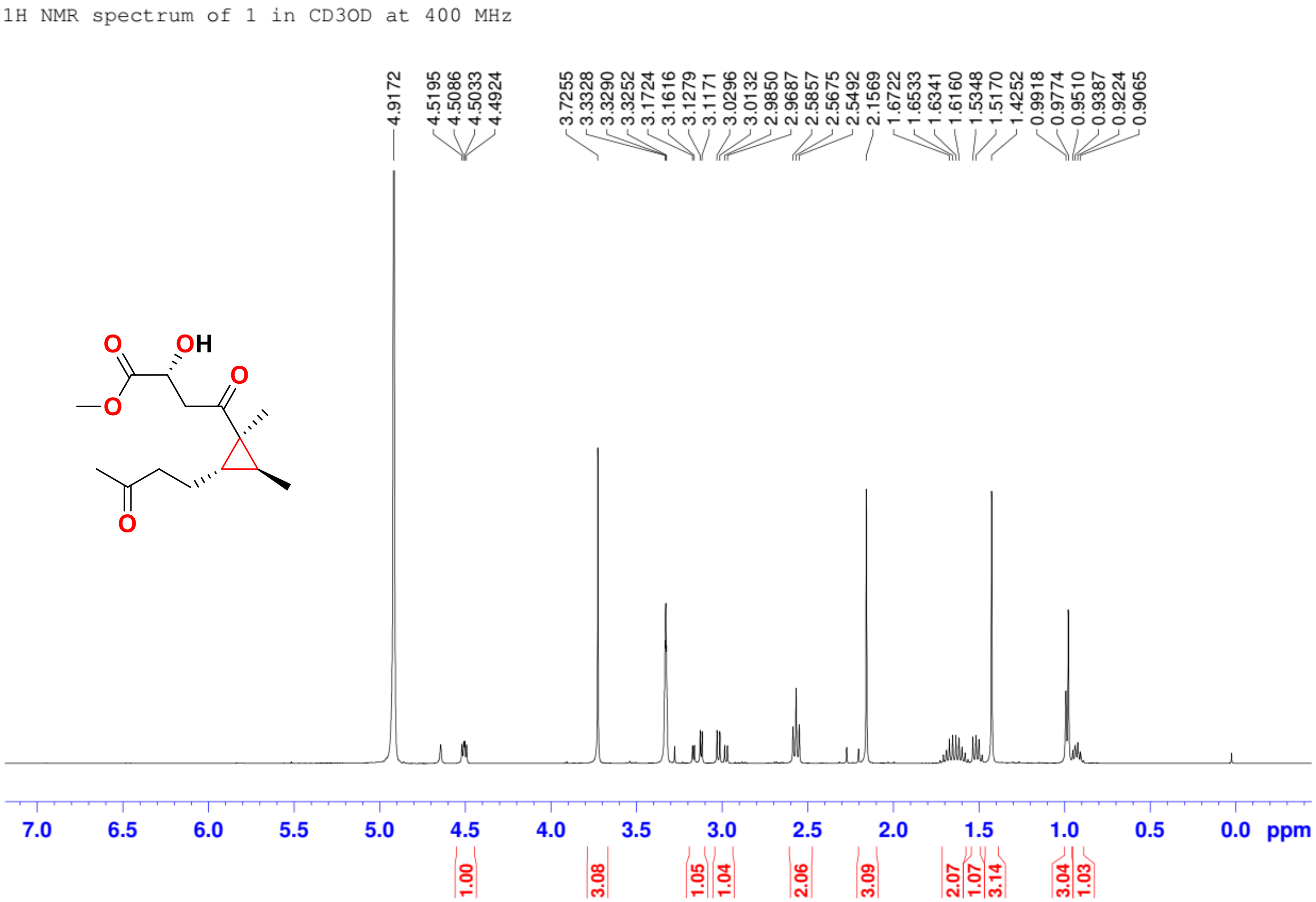

Figure S2. ^{1}H NMR spectrum of **1** in CD_3OD (400 MHz).

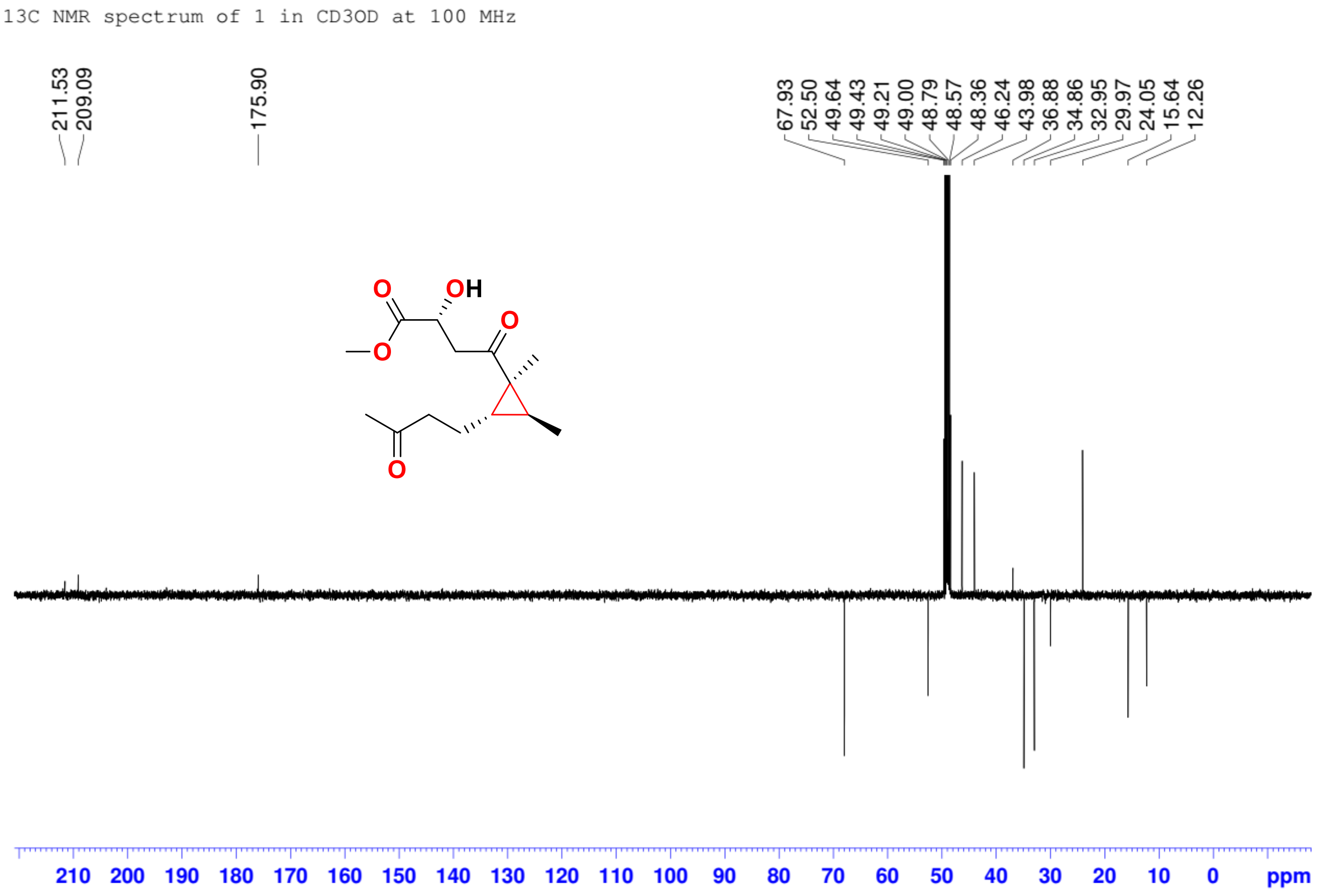

Figure S3. ^{13}C NMR spectrum of **1** in CD_3OD (100 MHz).

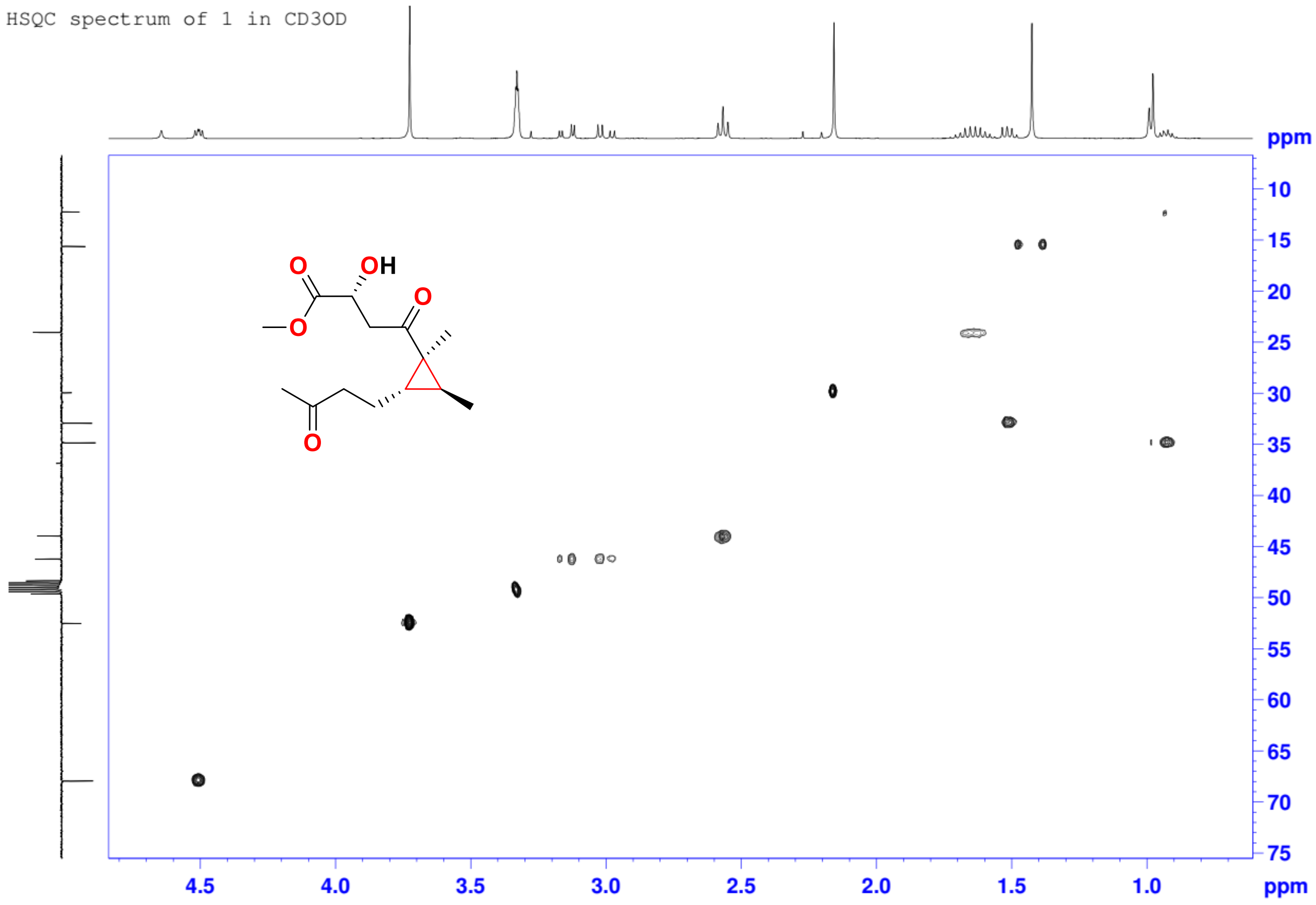

Figure S4. HSQC spectrum of **1** in CD_3OD.

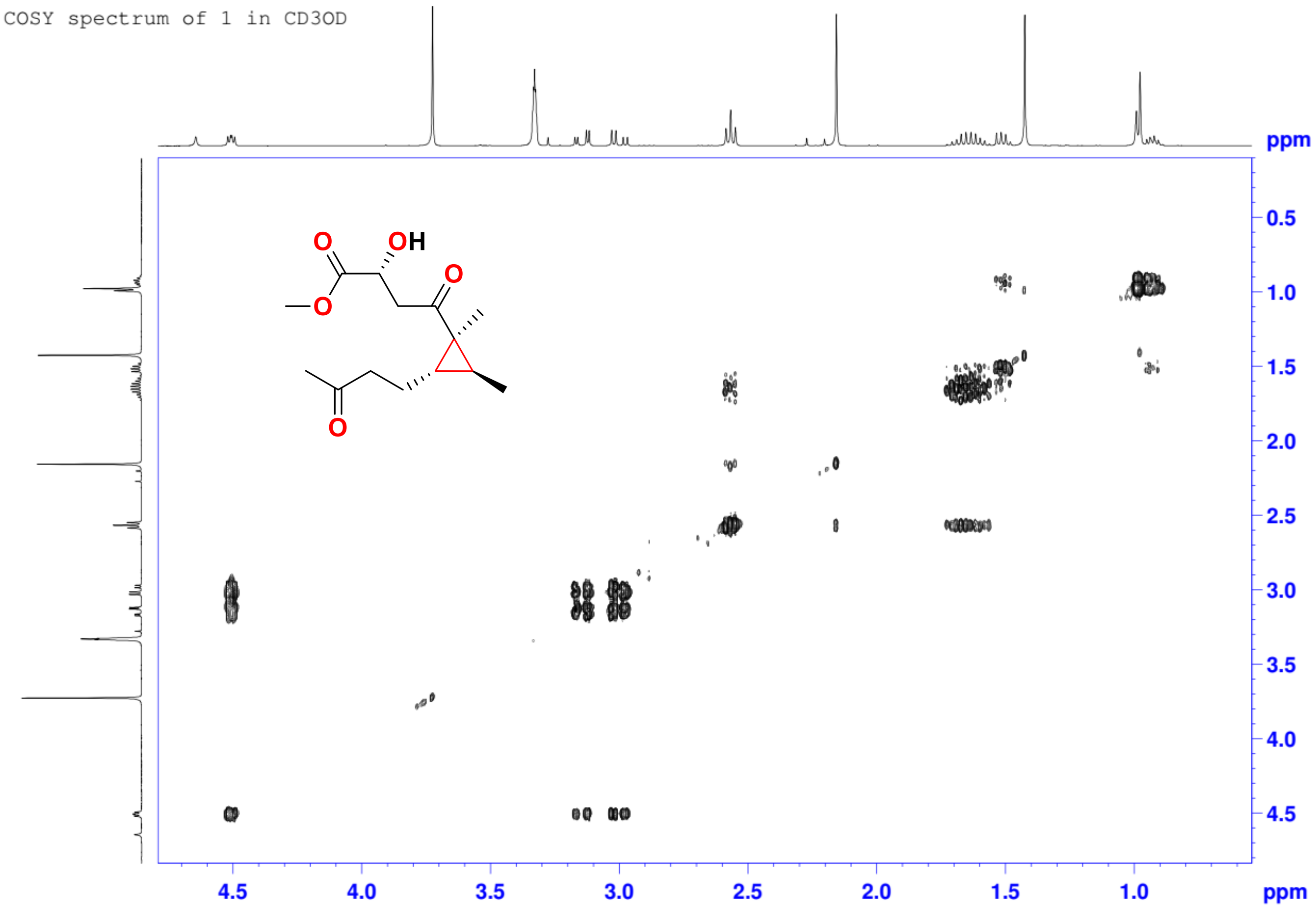

Figure S5. COSY spectrum of **1** in CD_3OD.

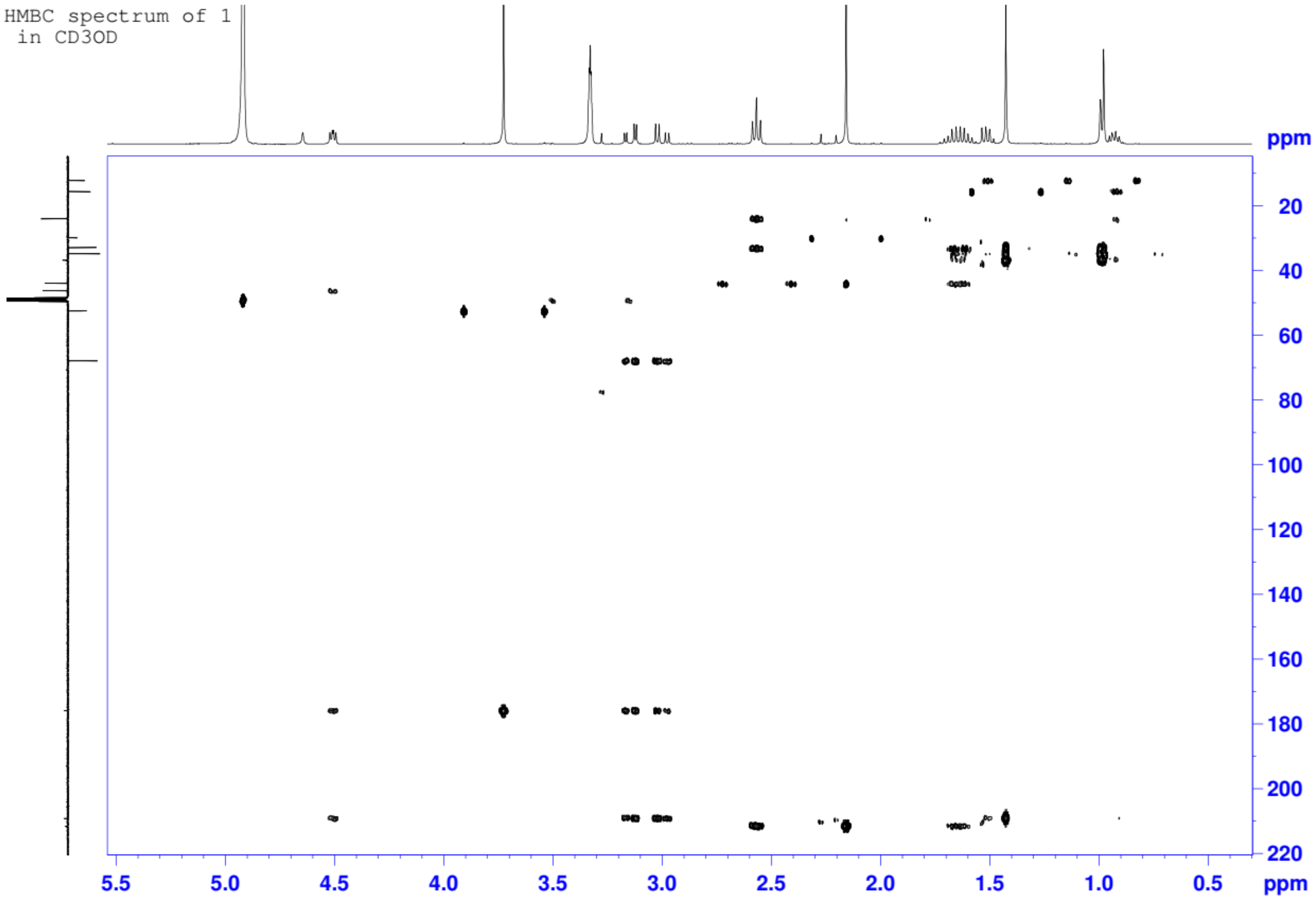

Figure S6. HMBC spectrum of **1** in CD_3OD.

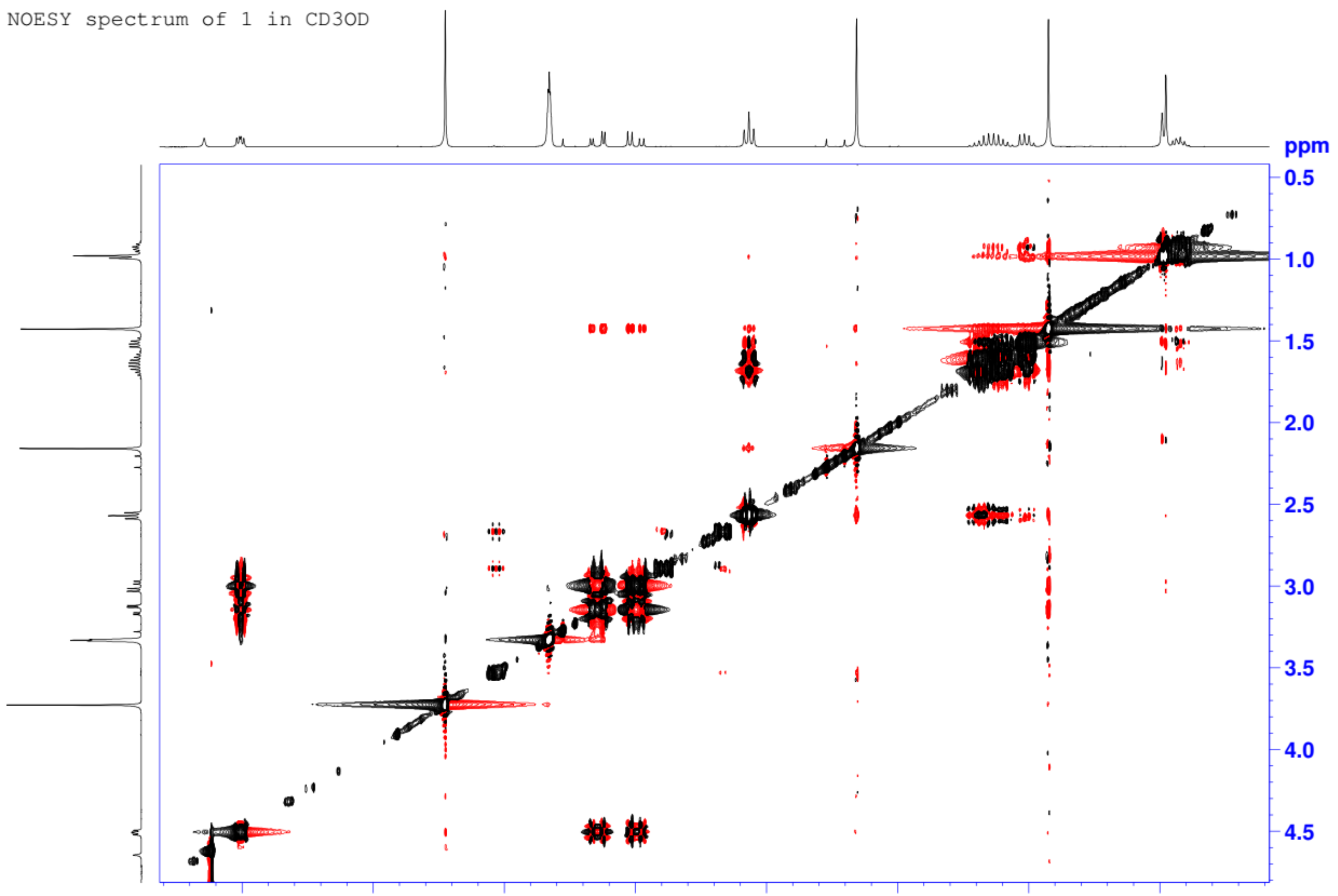

Figure S7. NOESY spectrum of **1** in CD_3OD.

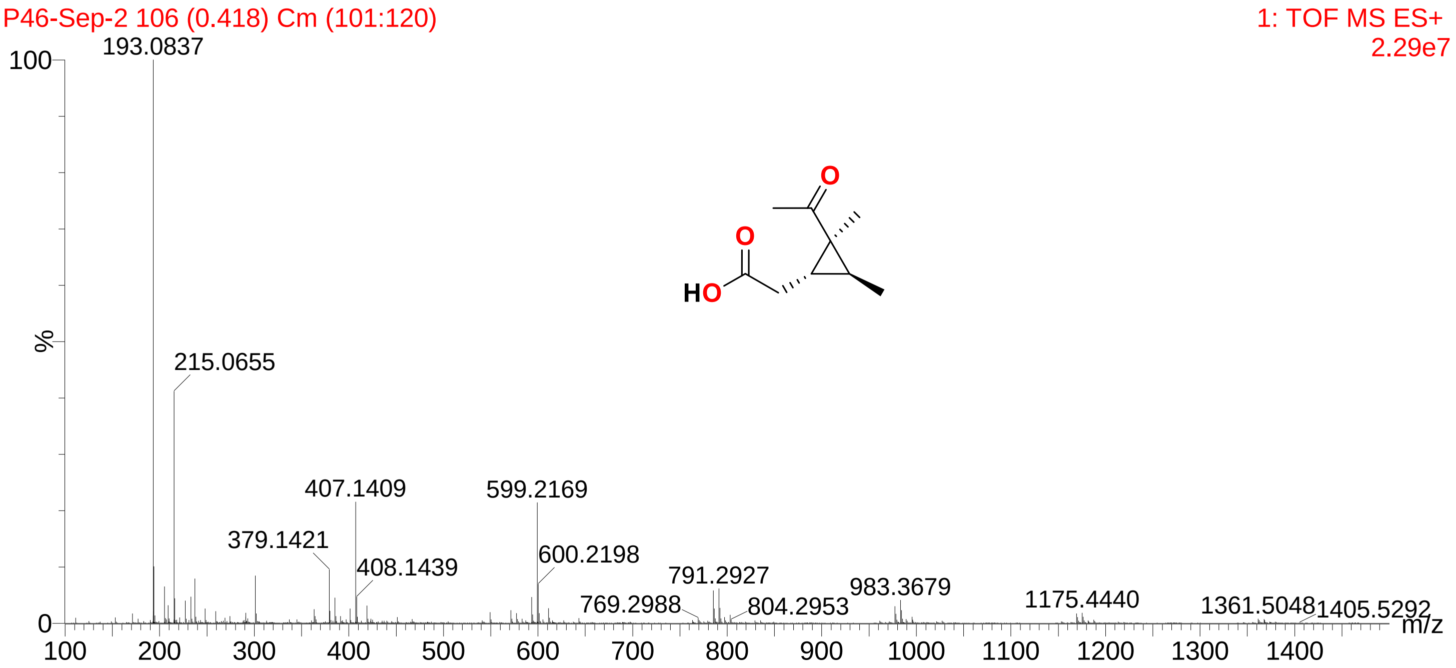

Figure S8. HRESIMS spectrum of **2**.

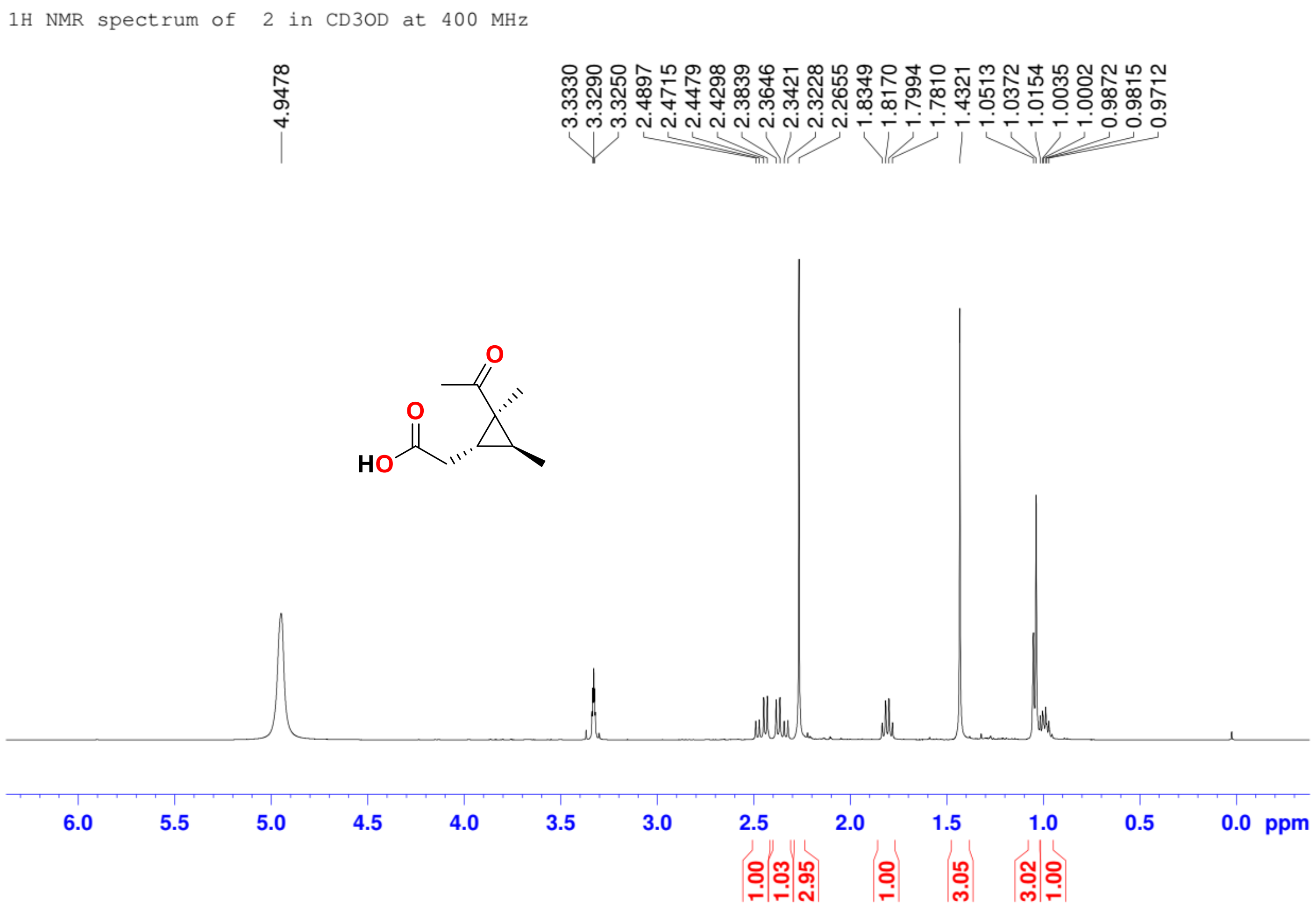

Figure S9. ^{1}H NMR spectrum of **2** in CD_3OD (400 MHz).

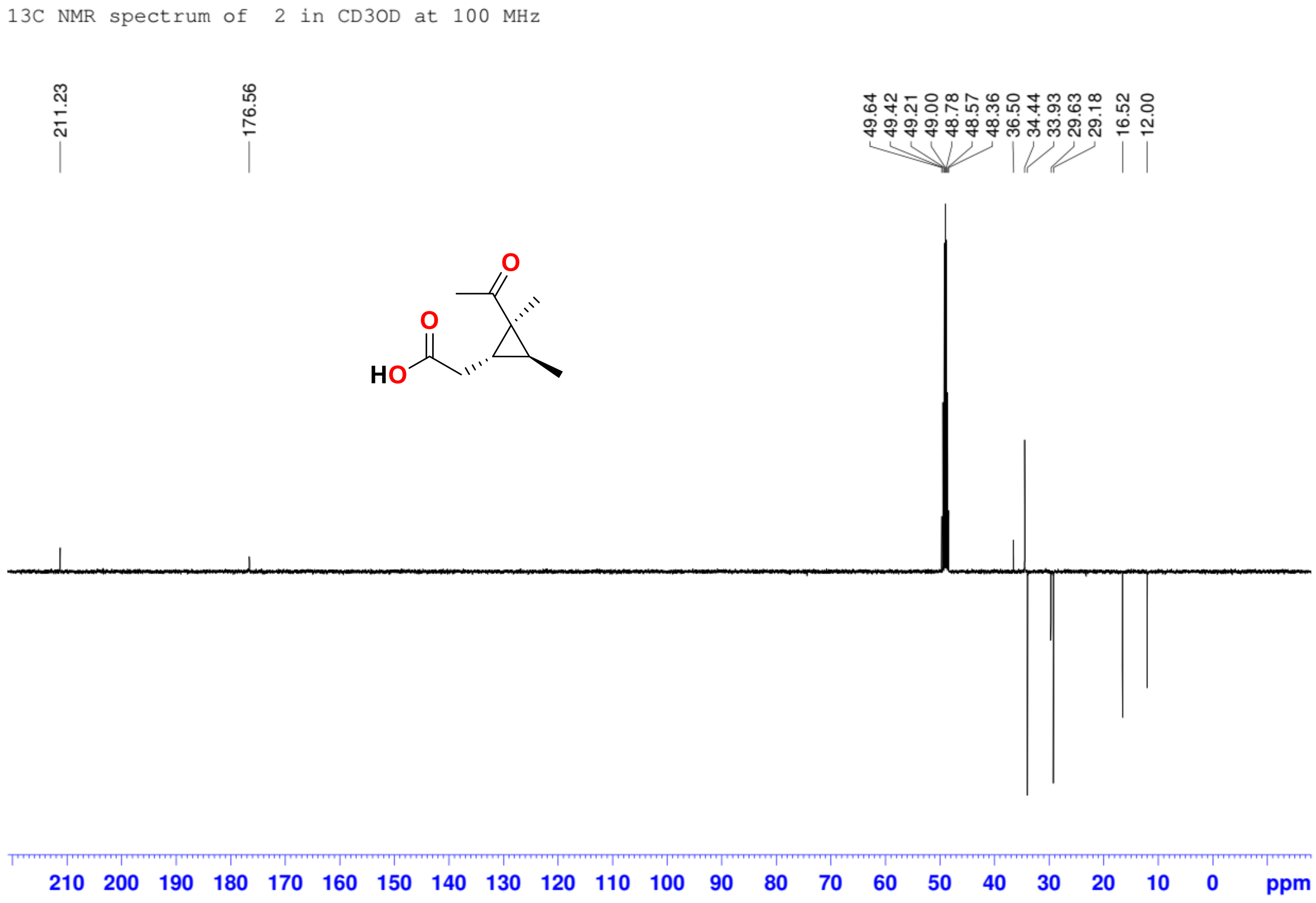

Figure S10. ^{13}C NMR spectrum of **2** in CD_3OD (100 MHz).

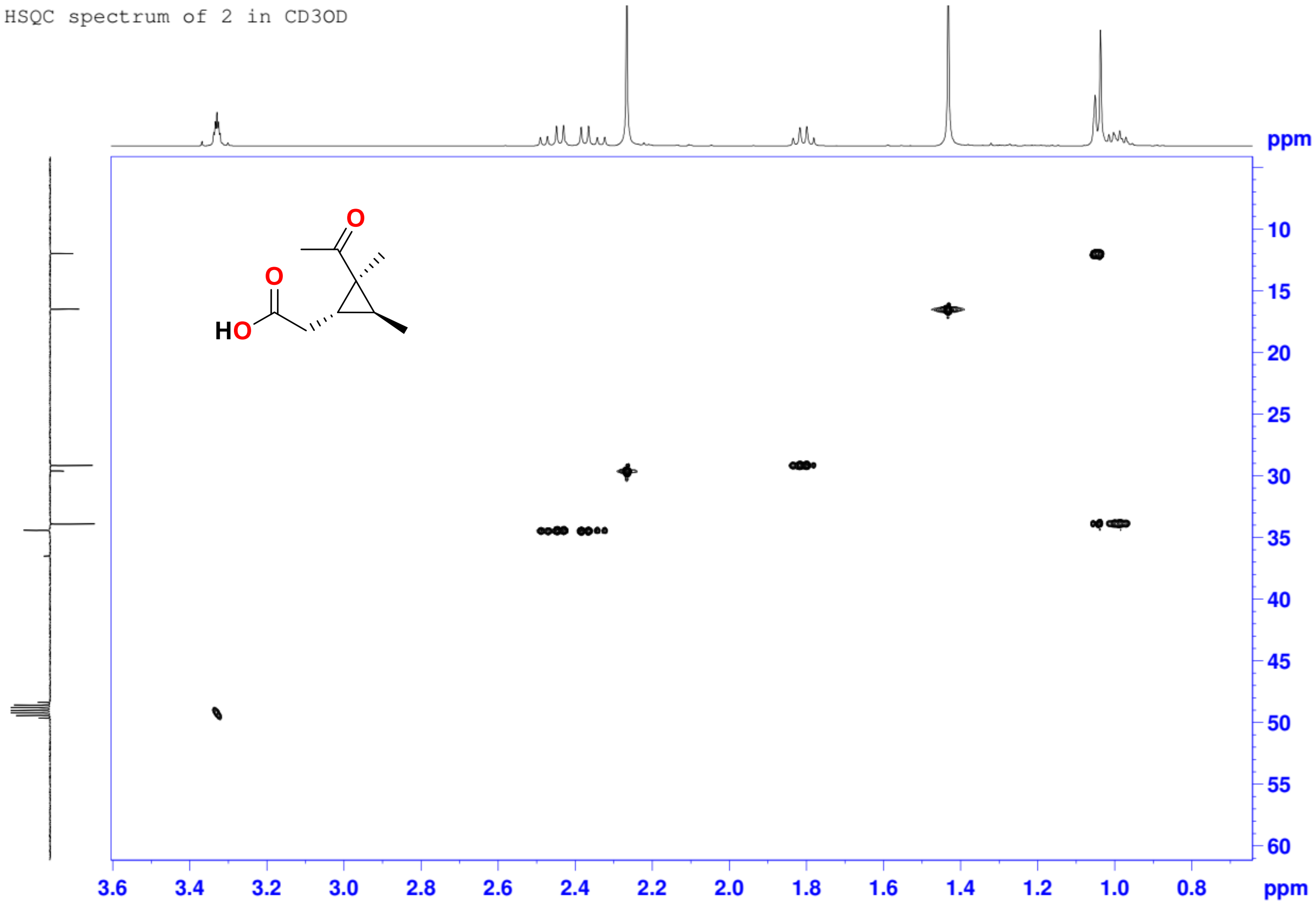

Figure S11. HSQC spectrum of **2** in CD_3OD.

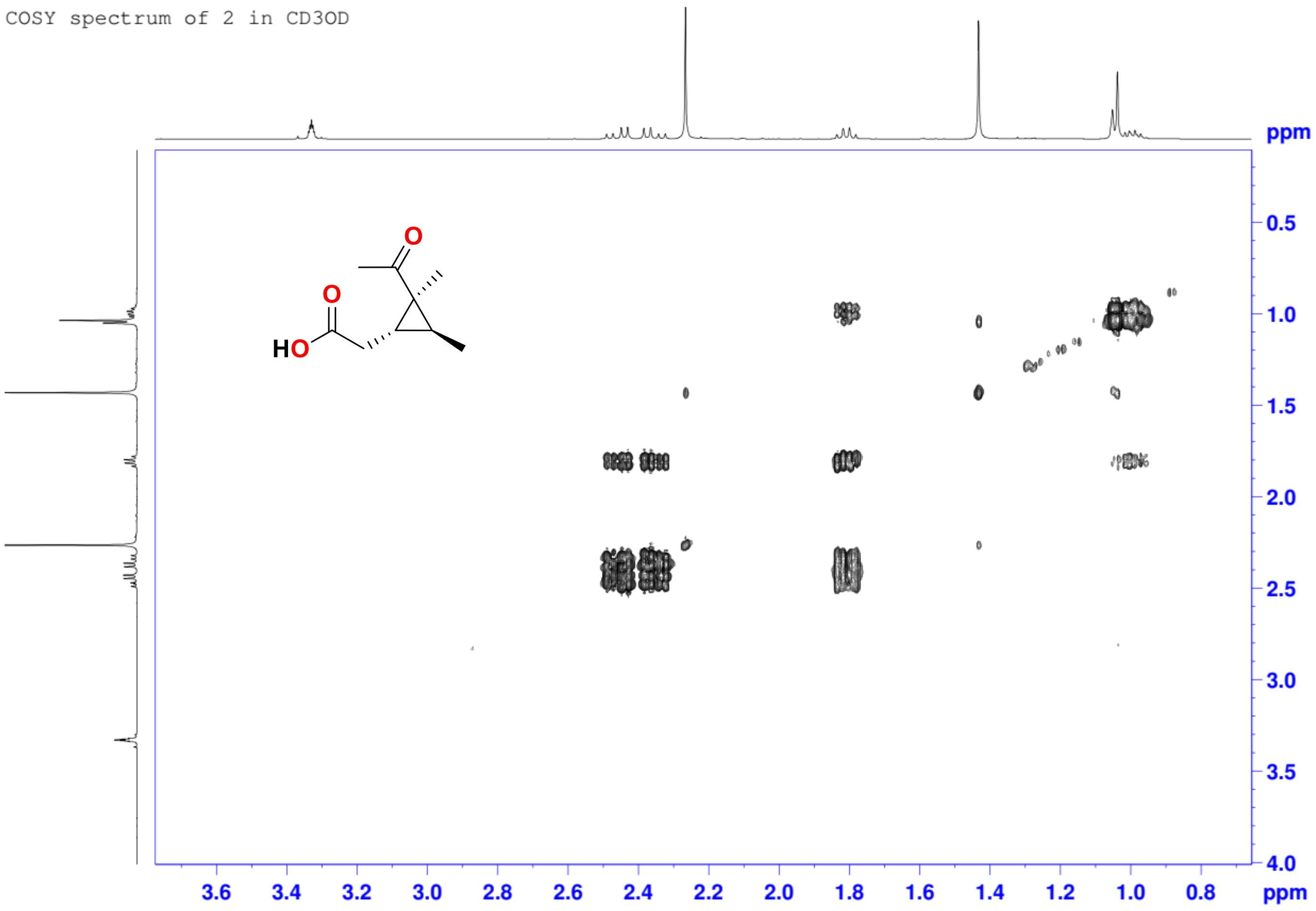

Figure S12. COSY spectrum of **2** in CD_3OD.

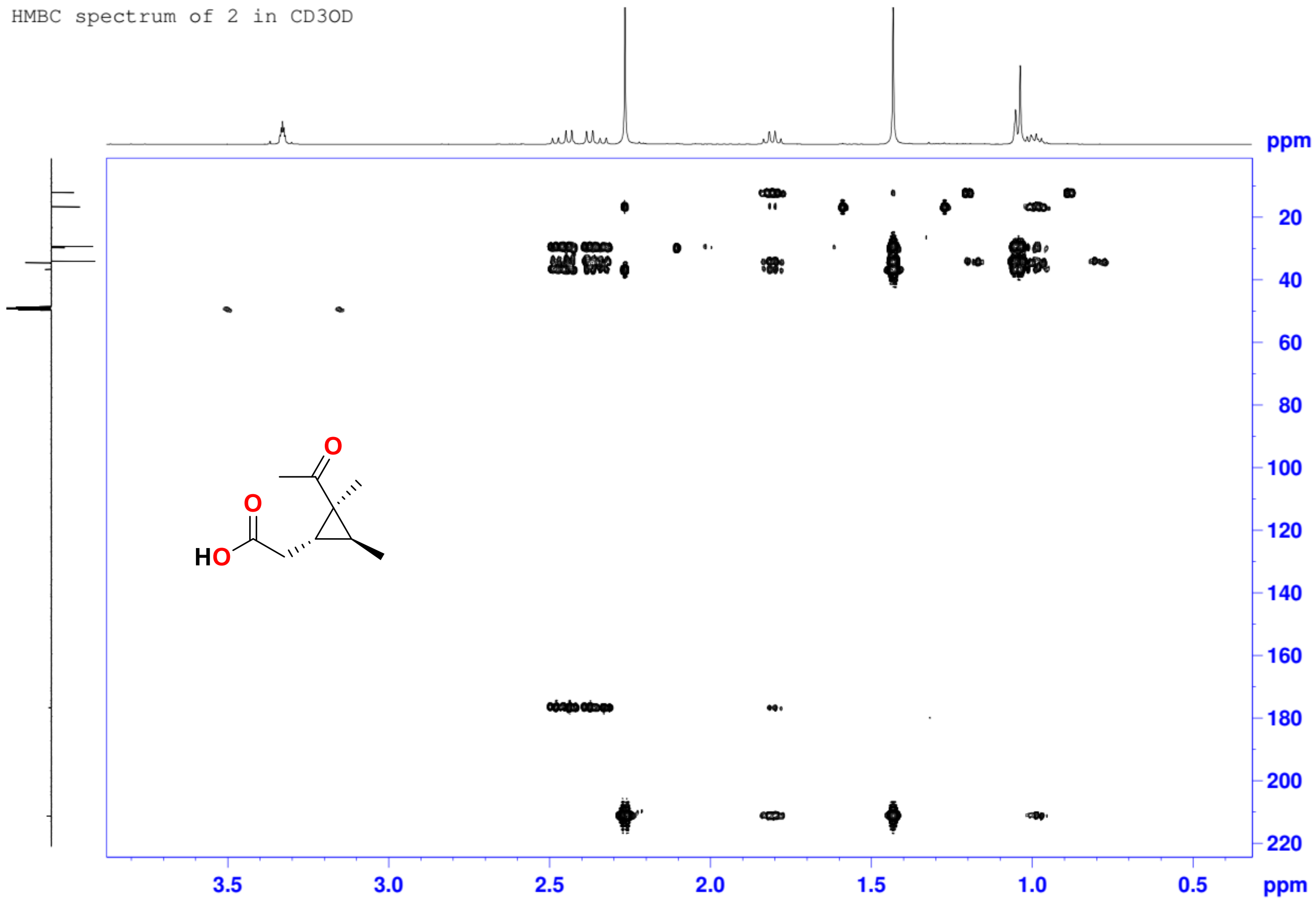

Figure S13. HMBC spectrum of **2** in CD_3OD.

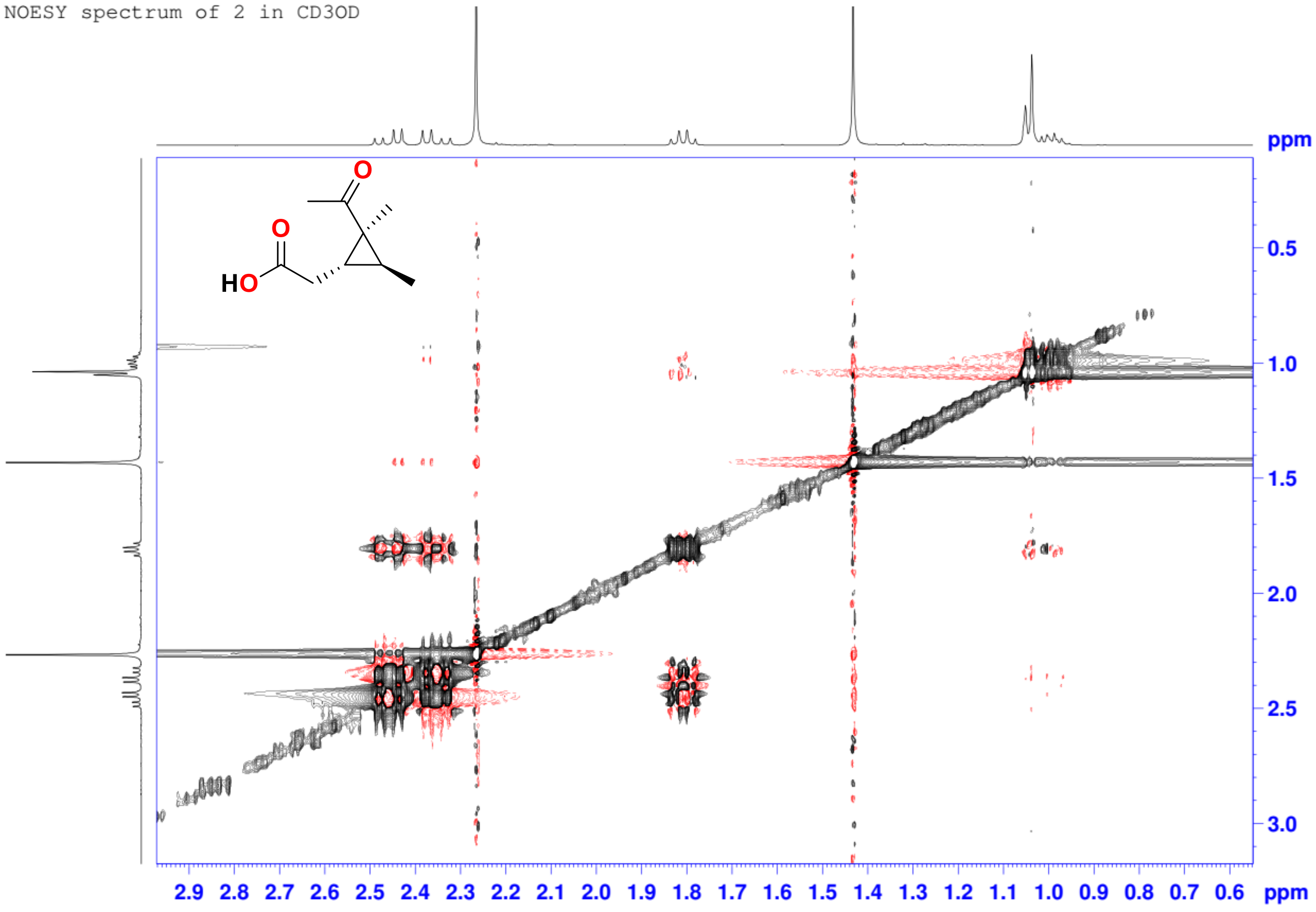

Figure S14. NOESY spectrum of **2** in CD_3OD.

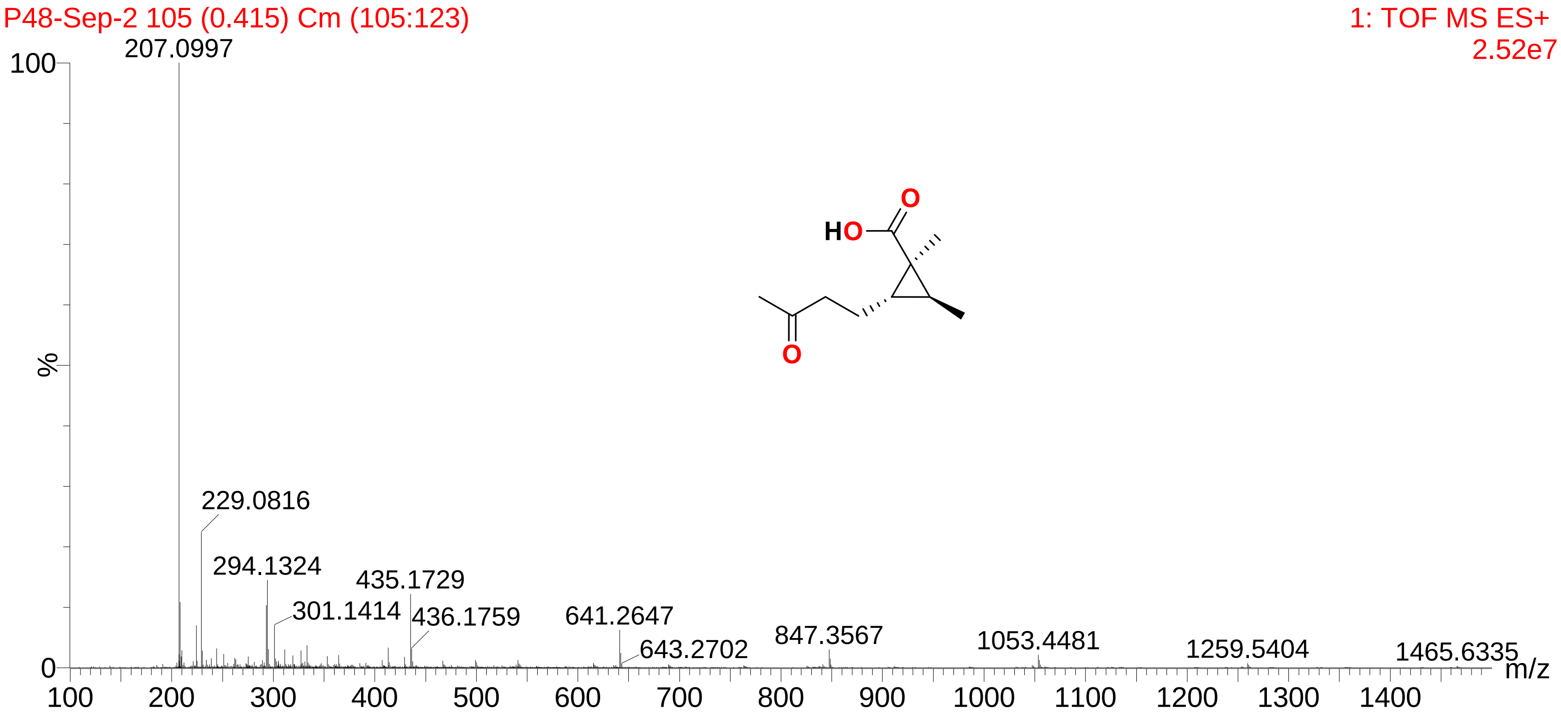

Figure S15. HRESIMS spectrum of **3**.

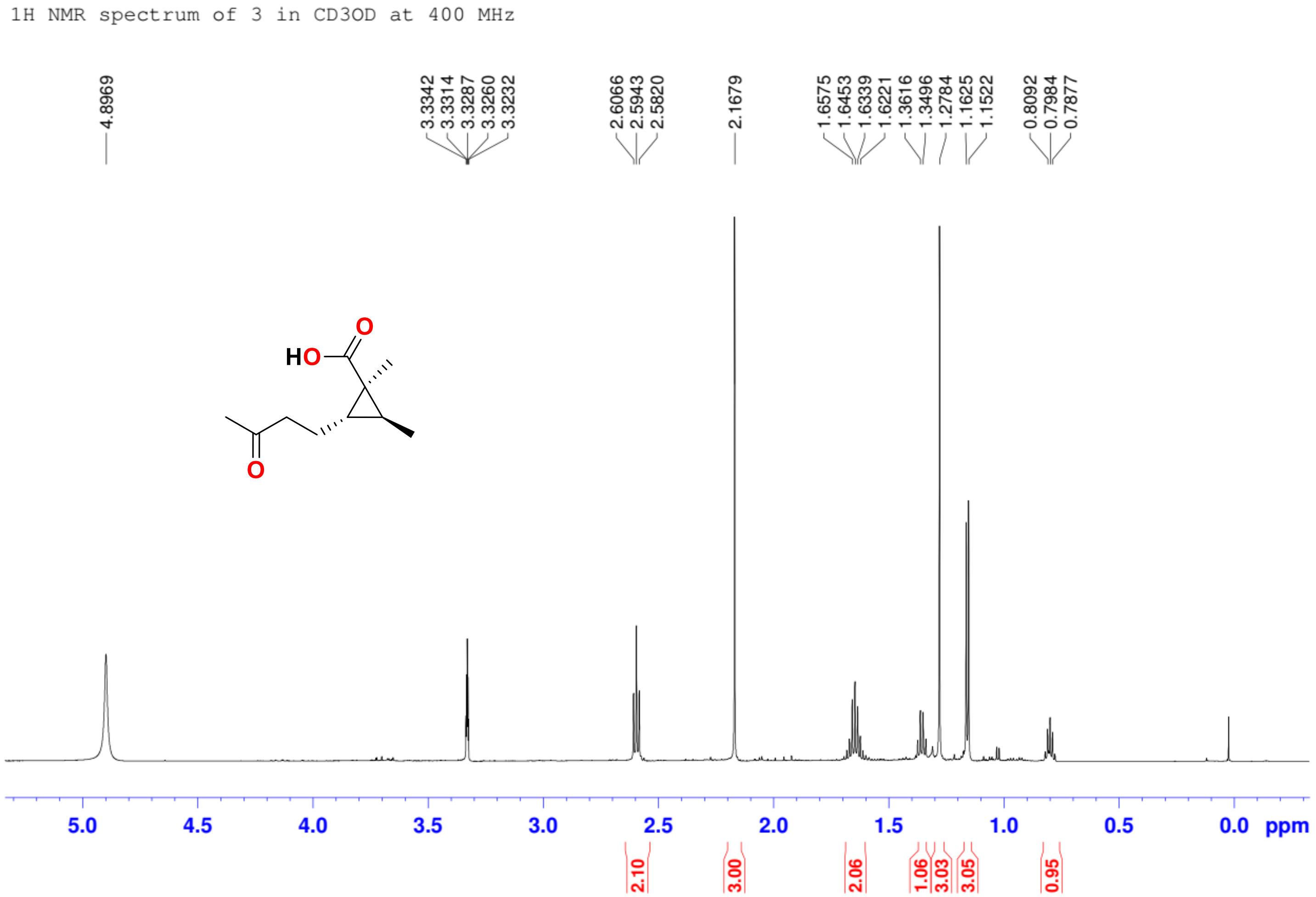

Figure S16. ¹H NMR spectrum of **3** in CD_3OD (400 MHz).

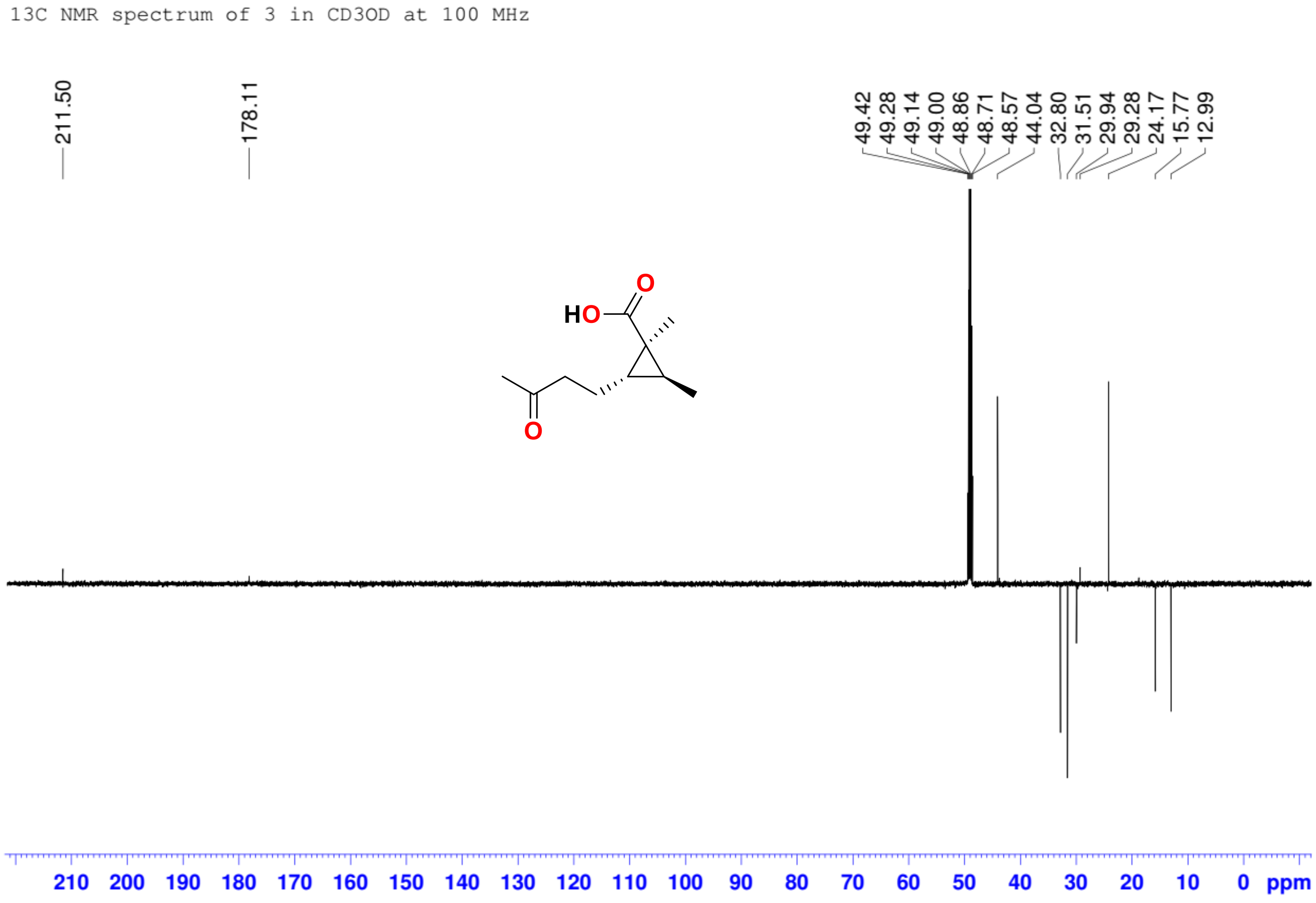

Figure S17. ^{13}C NMR spectrum of **3** in CD_3OD (100 MHz).

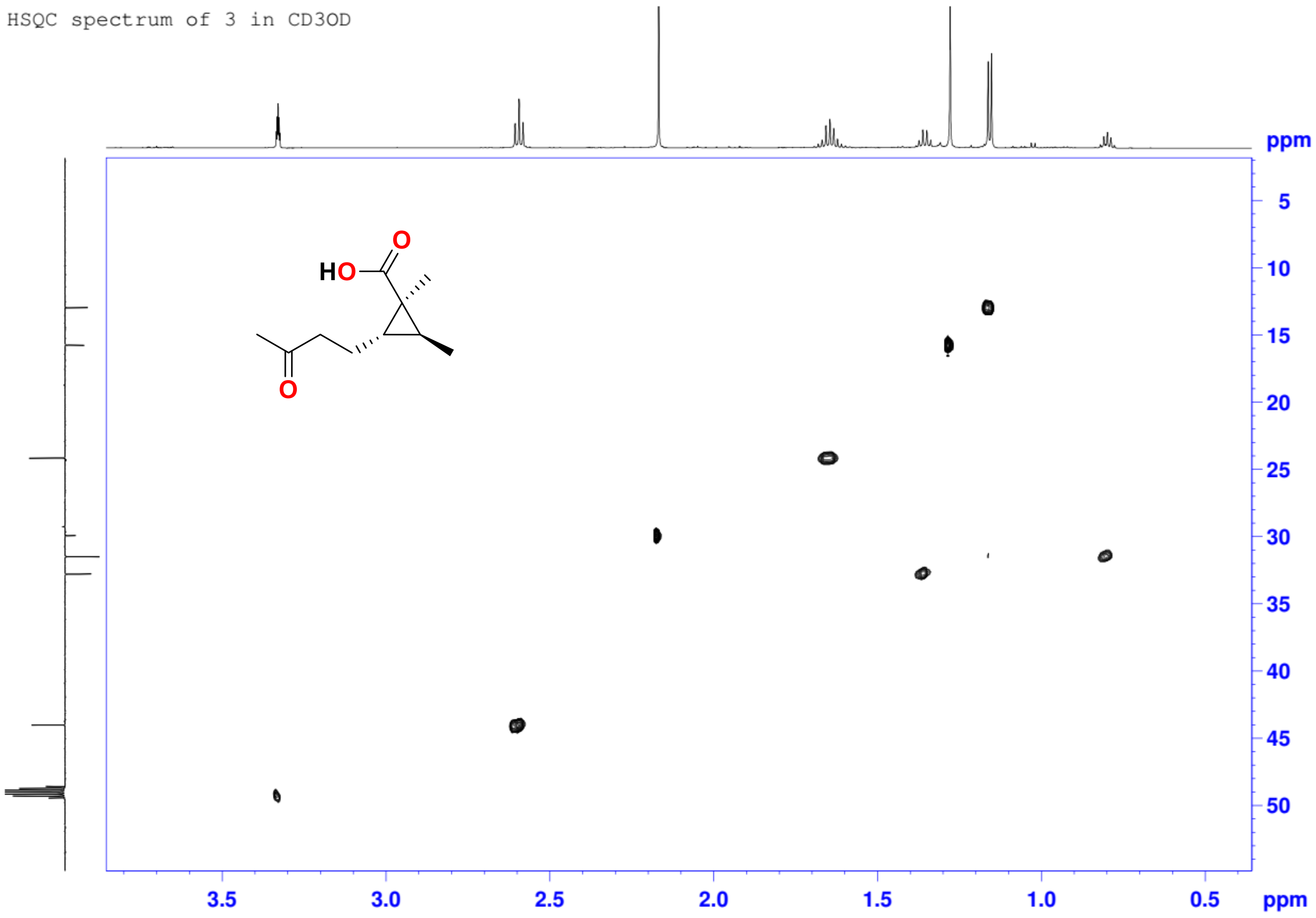

Figure S18. HSQC spectrum of **3** in CD_3OD.

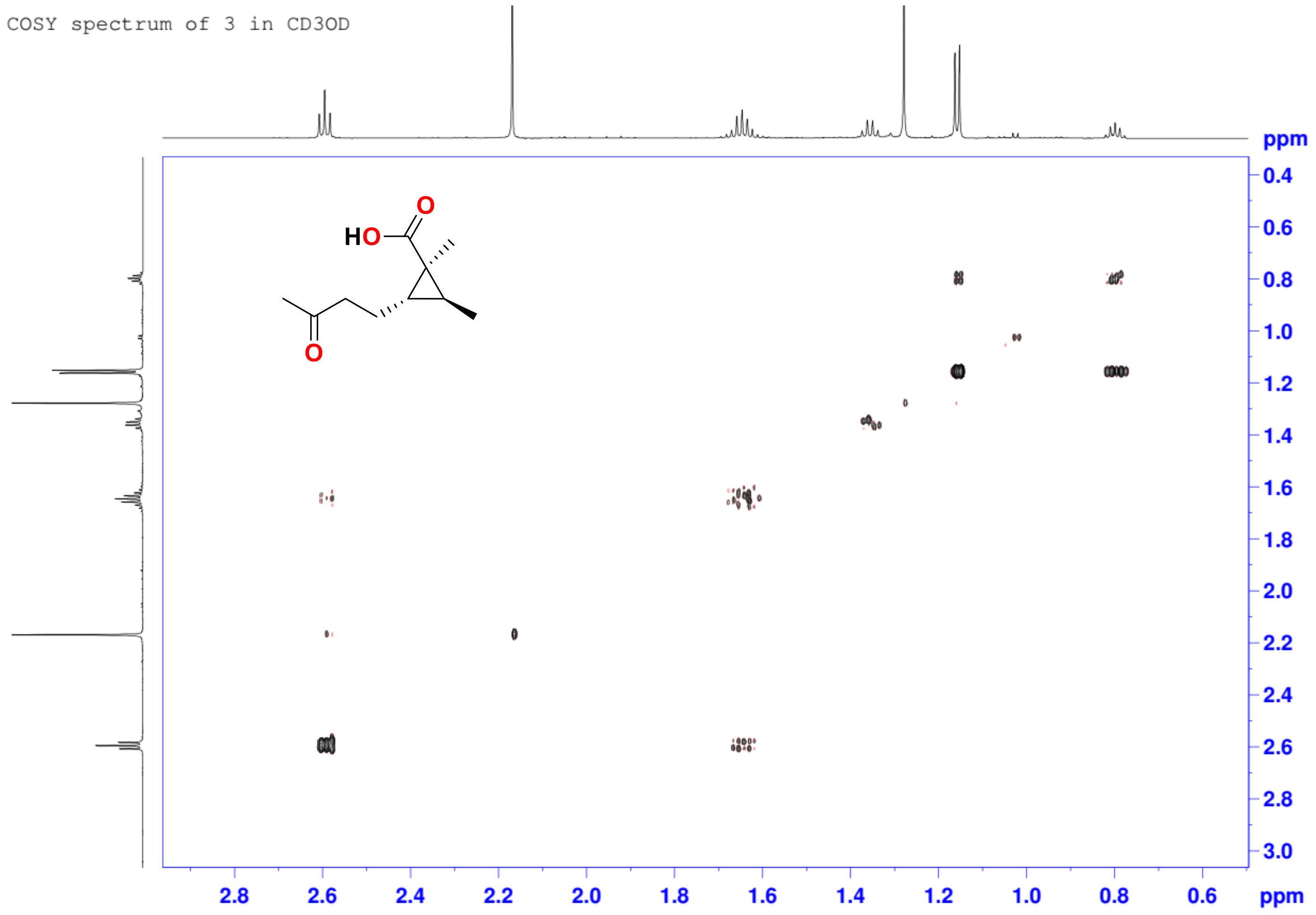

Figure S19. COSY spectrum of **3** in CD_3OD.

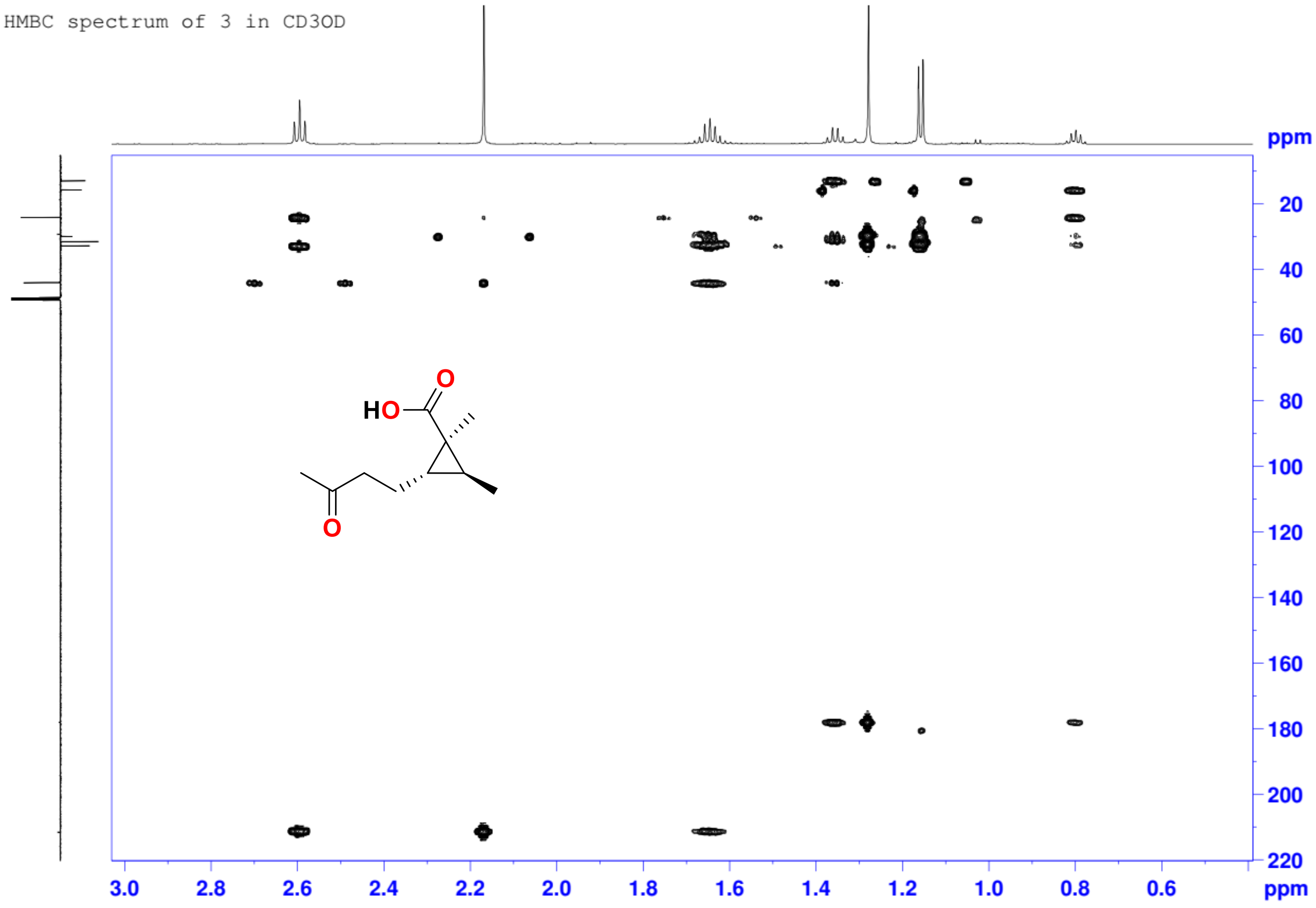

Figure S20. HMBC spectrum of **3** in CD_3OD.

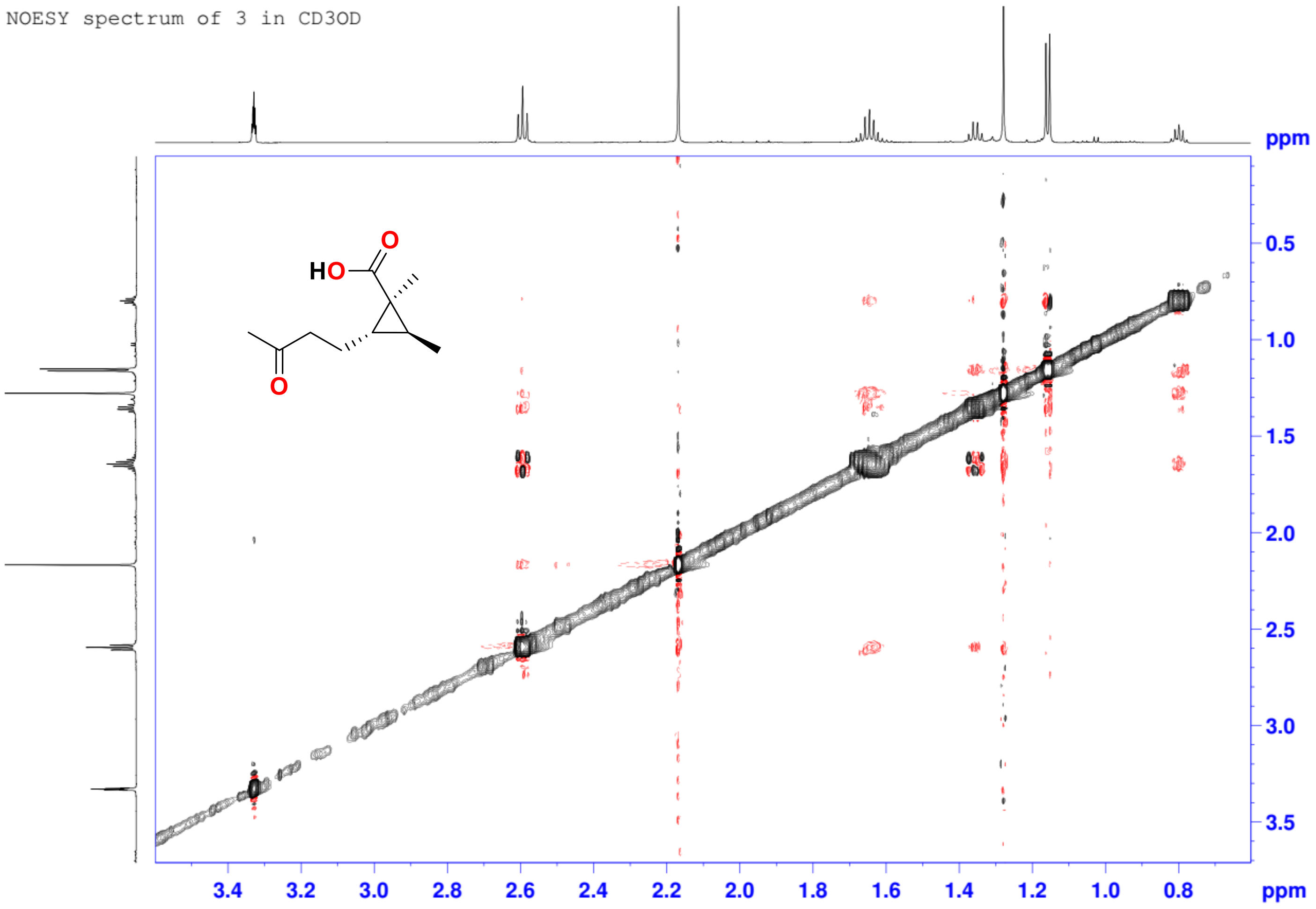

Figure S21. NOESY spectrum of **3** in CD_3OD.

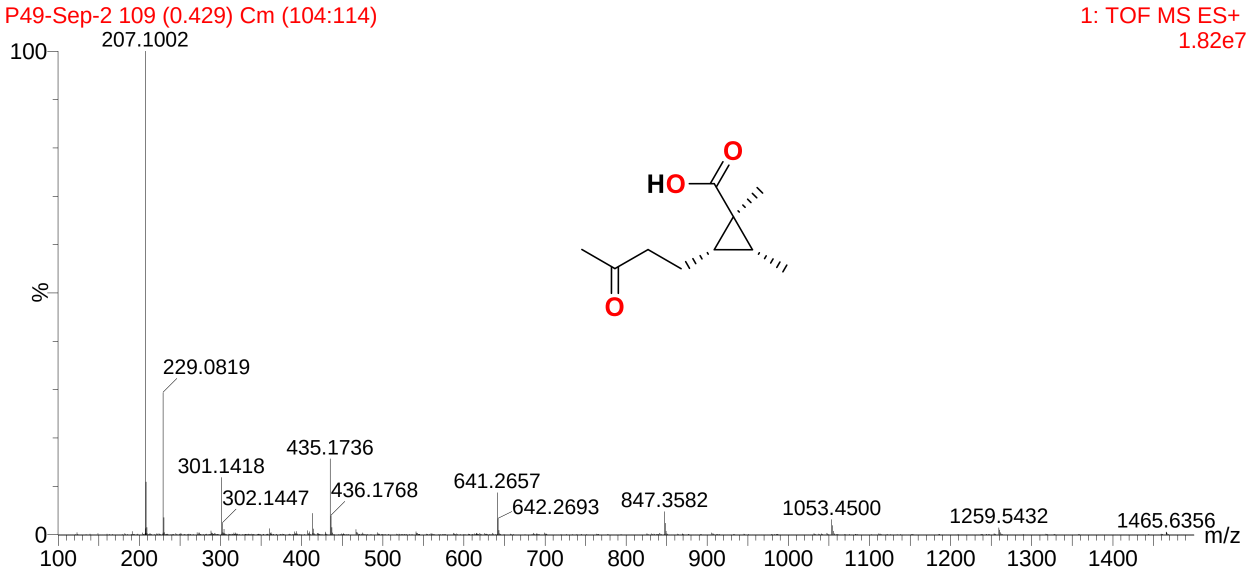

Figure S22. HRESIMS spectrum of **4**.

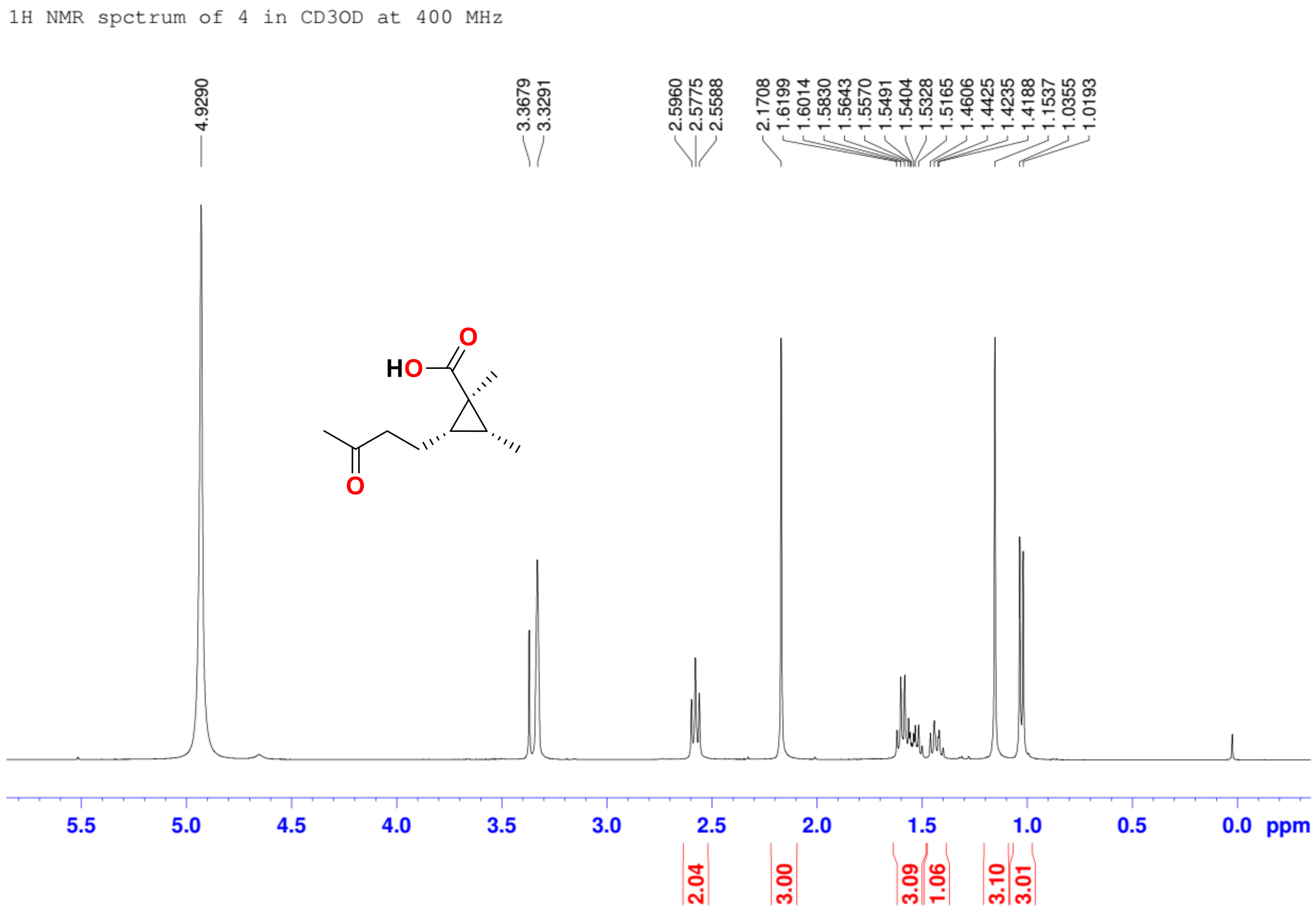

Figure S23. ^{1}H NMR spectrum of **4** inCD₃OD (400 MHz).

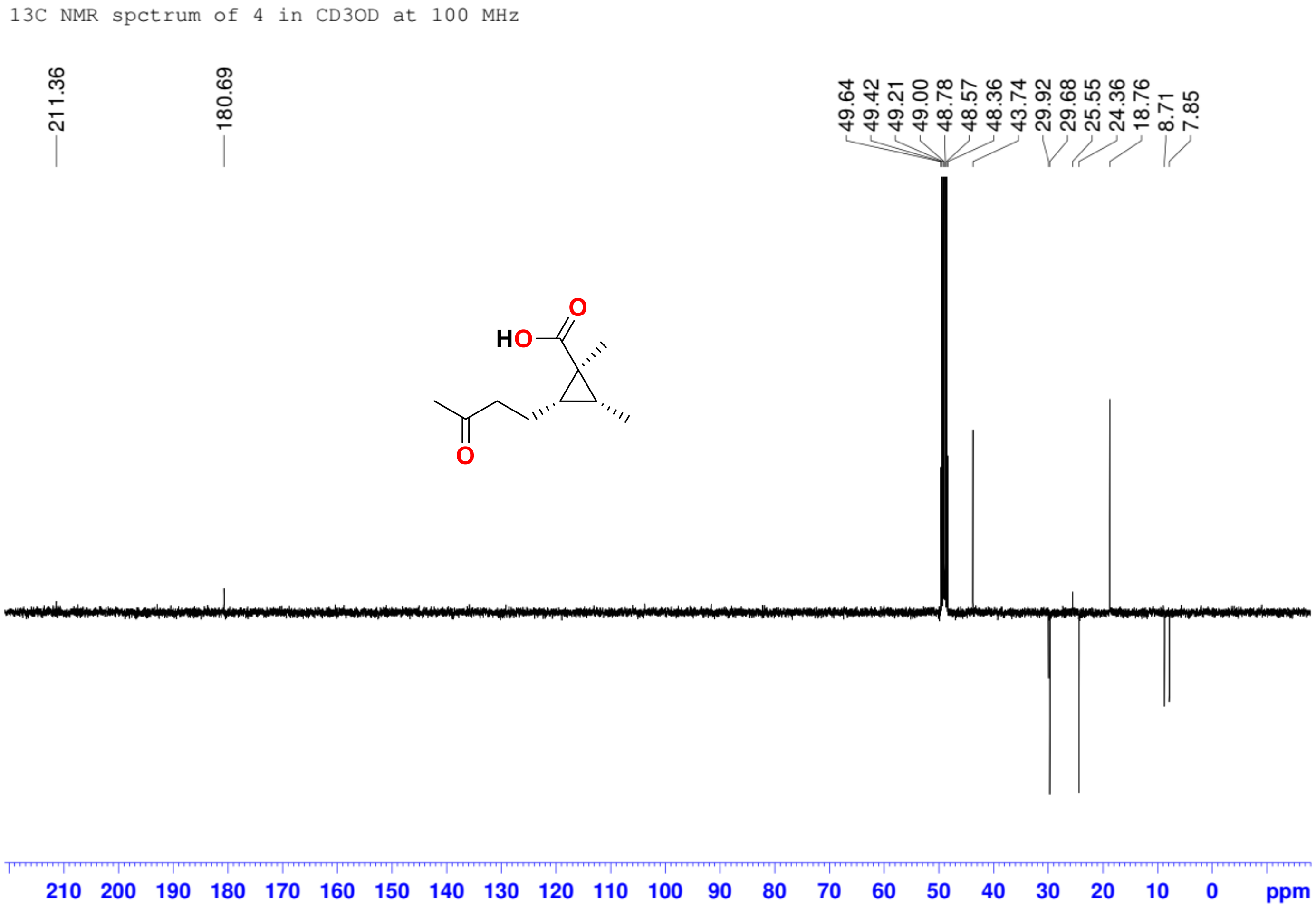

Figure S24. ^{13}C NMR spectrum of **4** in CD_3OD (100 MHz).

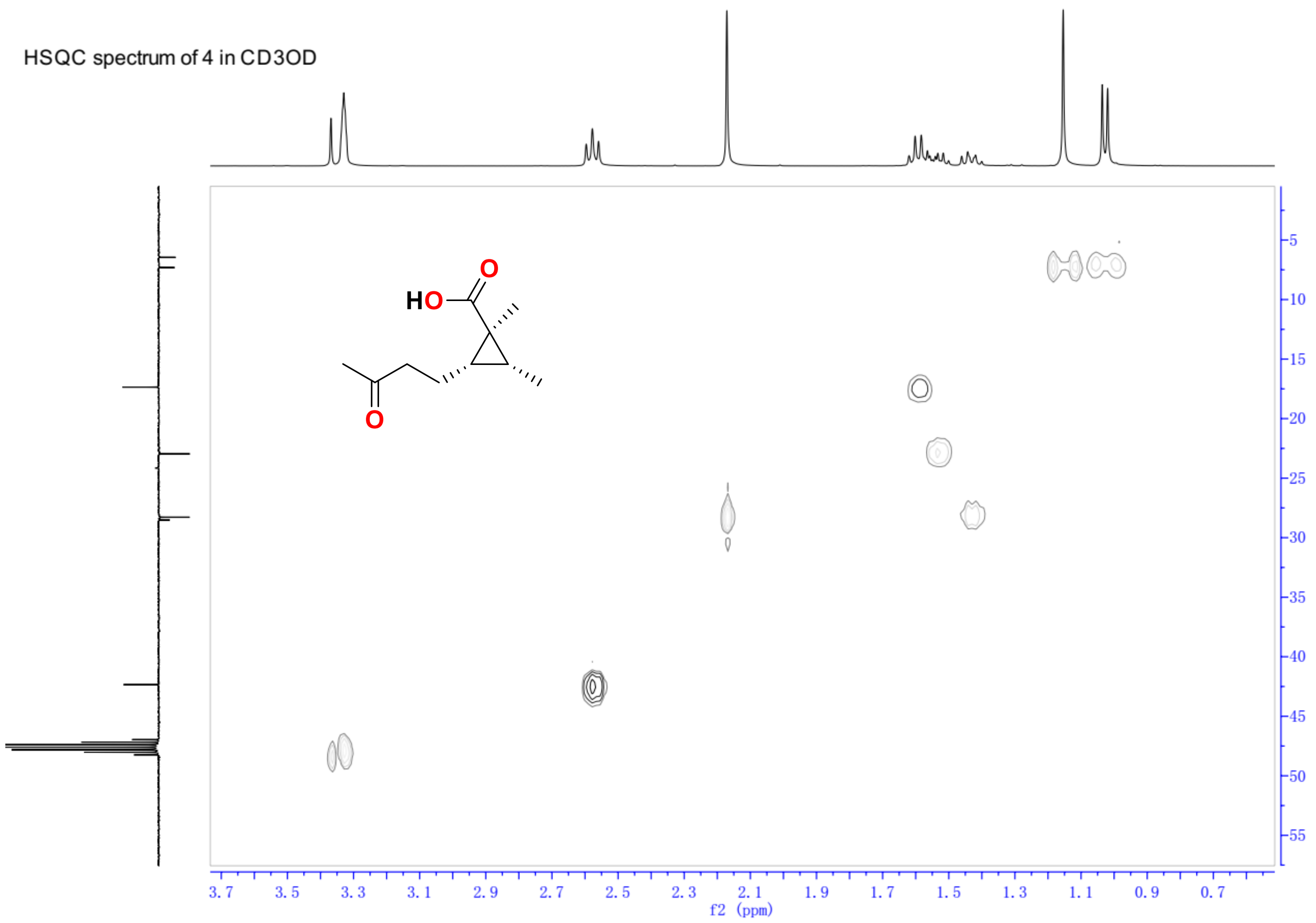

Figure S25. HSQC spectrum of **4** in CD_3OD.

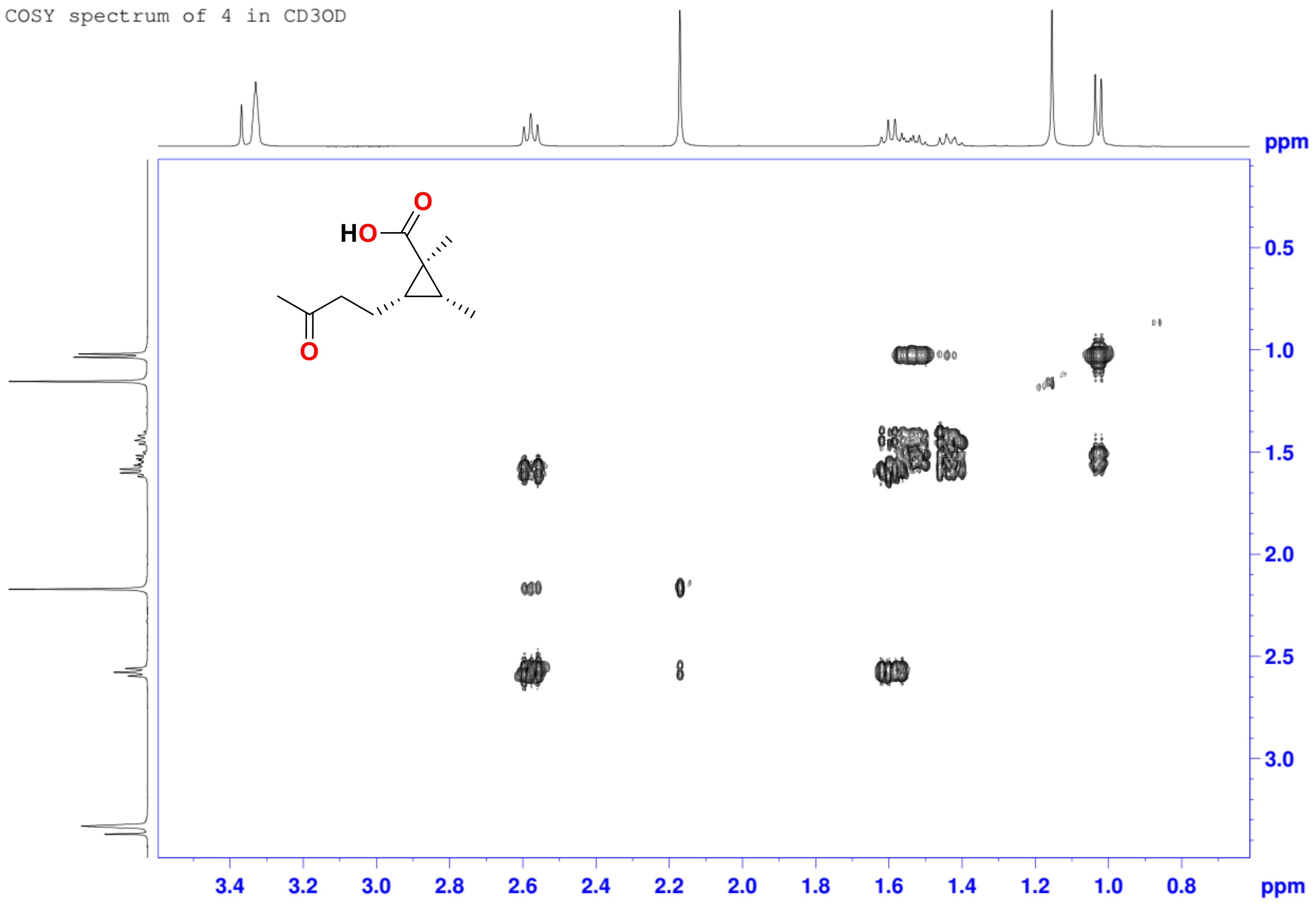

Figure S26. COSY spectrum of **4** inCD_3OD.

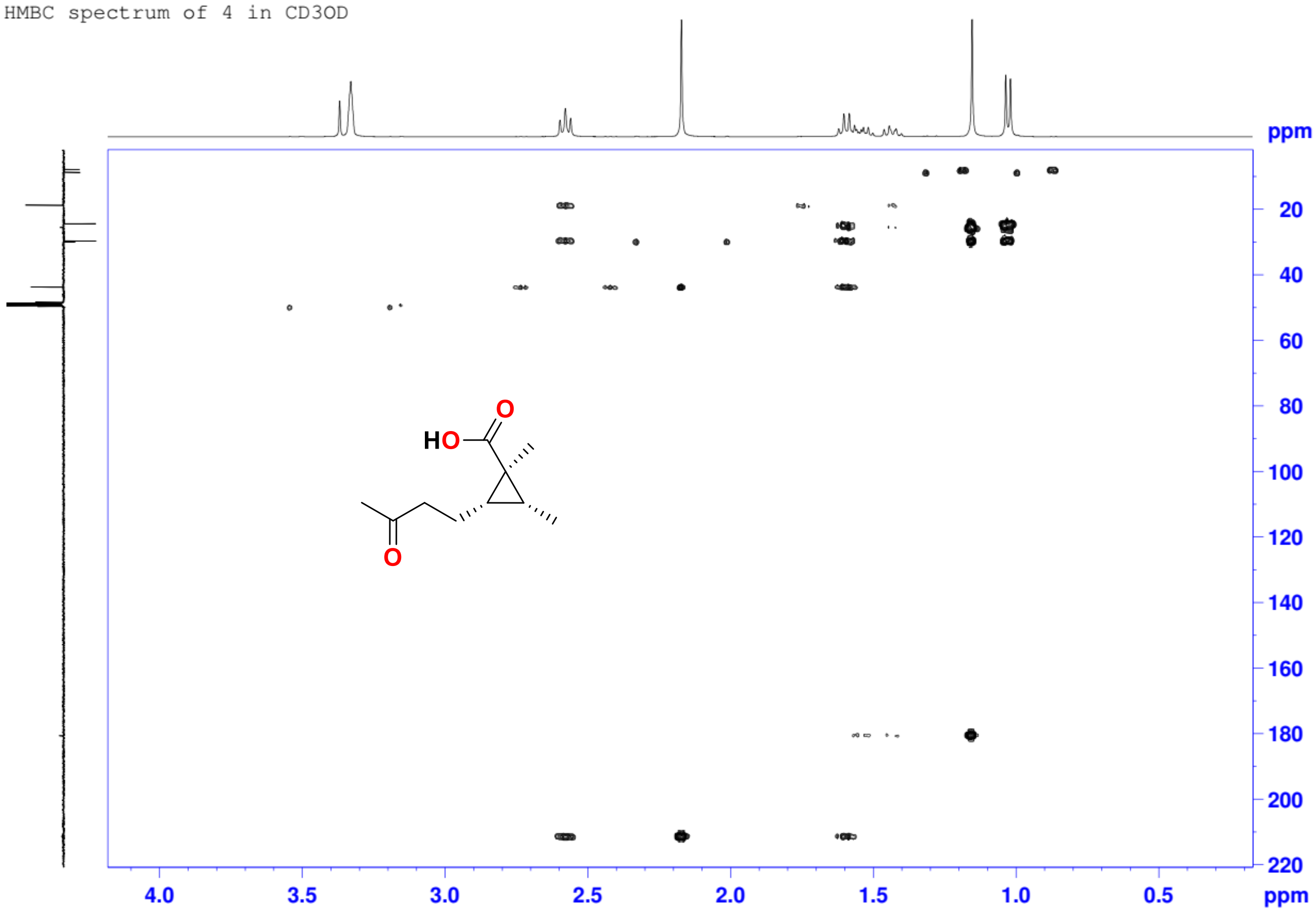

Figure S27. HMBC spectrum of **4** in CD_3OD.

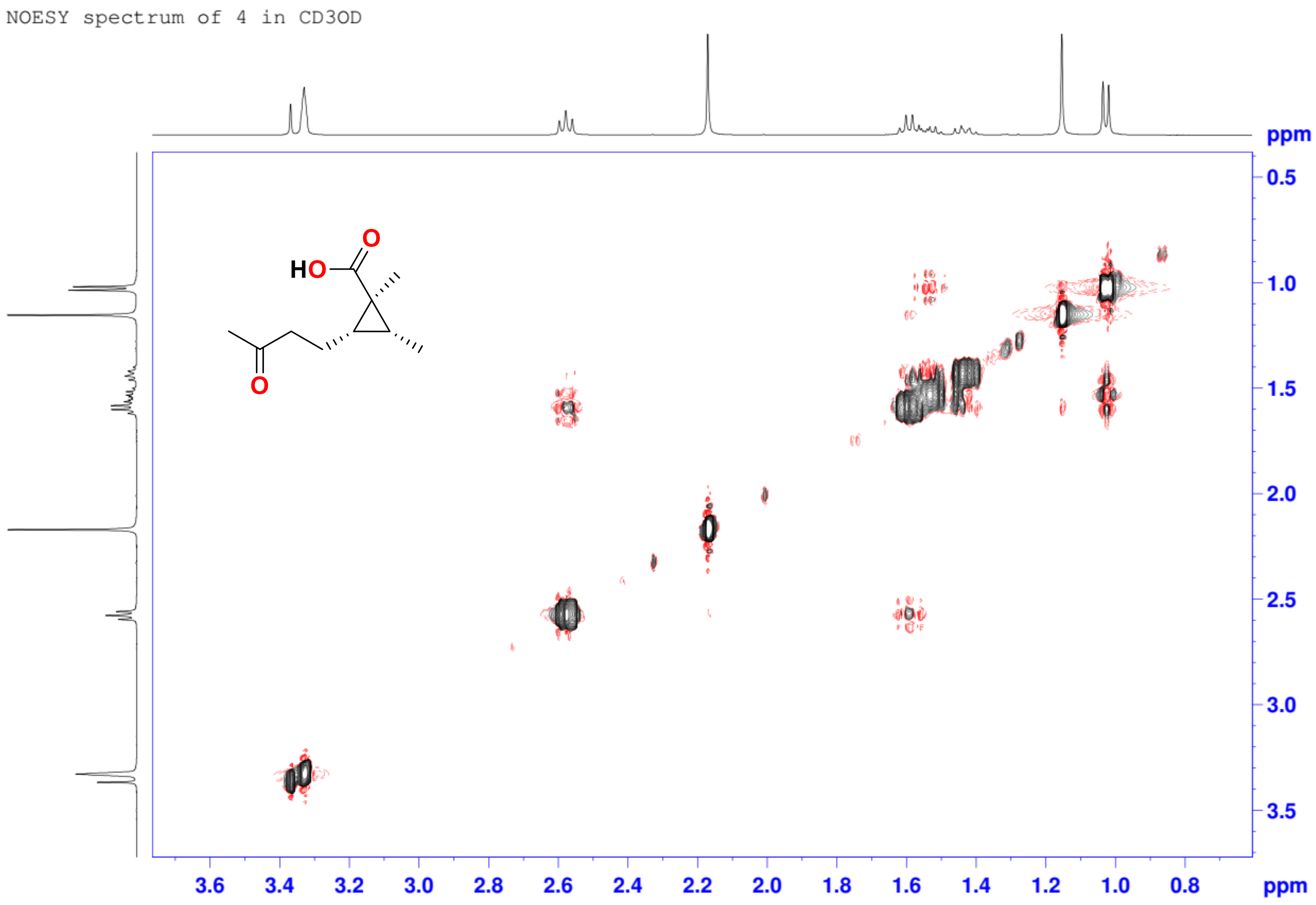

Figure S28. NOESY spectrum of **4** in CD_3OD.

Functional		Solvent?		Basis Set		Type of Data	
mPW1PW91		PCM		6-31+G(d,p)		Shielding Tensors	
		DP4+	100.00%	0.00%	-	-	-
Nuclei	sp2?	Experimenta	Isomer 1	Isomer 2	Isomer 3	Isomer 4	Isomer 5
C		36.9	153.9276519	151.5862828			
C		33	154.097521	147.6021002			
C		34.9	155.4414868	149.7295482			
C		12.3	183.7727846	179.6965875			
C	x	209.1	-16.9854338	-20.0519483			
C		15.6	178.2658593	174.131417			
C		46.2	147.8840458	145.7561892			
C		67.9	123.0161635	120.0503683			
C	x	175.9	21.70139936	20.98974957			
C		24.1	171.2997154	166.4754922			
C		44	150.5873307	148.3650511			
C	x	211.5	-15.4213583	-13.1996784			
C		30	164.5948938	160.6139202			
C		52.5	143.0804422	139.3401825			
H		1.52	30.19845931	29.31504335			
H		0.92	30.42200183	29.58995308			
H		0.98	30.77338838	29.55655509			
H		0.98	30.49002123	29.59461985			
H		0.98	30.48398777	29.89388943			
H		1.43	29.49876604	29.56513725			
H		1.43	30.00205949	29.10925637			
H		1.43	30.57549646	29.78859647			
H		3	28.78534999	28.21942111			
H		3.15	28.03118132	27.20745666			
H		4.51	27.10173952	26.63969409			
H		1.64	30.0240035	29.66891557			
H		1.64	29.86446886	28.71522862			
H		2.57	28.92798665	28.1555014			
H		2.57	28.8045336	28.3454392			
H		2.16	29.43418685	28.61682517			
H		2.16	29.14476762	28.51033101			
H		2.16	29.18618679	28.33097572			
H		3.73	27.78104886	27.18871343			
H		3.73	27.66236435	27.06212118			
H		3.73	27.78420645	27.06566204			

Functional	Solvent?		Basis Set		Type of Data	
mPW1PW91	PCM		6-31+G(d,p)		Shielding Tensors	
	Isomer 1	Isomer 2	Isomer 3	Isomer 4	Isomer 5	Isomer 6
sDP4+ (H data)	100.00%	0.00%	-	-	-	-
sDP4+ (C data)	99.99%	0.01%	-	-	-	-
sDP4+ (all data)	100.00%	0.00%	-	-	-	-
uDP4+ (H data)	100.00%	0.00%	-	-	-	-
uDP4+ (C data)	100.00%	0.00%	-	-	-	-
uDP4+ (all data)	100.00%	0.00%	-	-	-	-
DP4+ (H data)	100.00%	0.00%	-	-	-	-
DP4+ (C data)	100.00%	0.00%	-	-	-	-
DP4+ (all data)	100.00%	0.00%	-	-	-	-

Figures S29. DP4+ probability analyses of (1*S*,2*S*,3*S*,12*R*)-**1** (isomer 1) and (1*S*,2*S*,3*S*,12*S*)-**1** (isomer 2) at the mPW1PW91/6-31+G(d,p) level.
